# An updated estimate of the proportion of neurocysticercosis among people with epileptic seizures: a systematic review and Bayesian meta-analysis

**DOI:** 10.1186/s40249-026-01501-1

**Published:** 2026-09-10

**Authors:** Mohammad Shah Jalal, Orane Figuet, Katrina Di Bacco, Marine Hubert, Sophie Adame, Dominik Stelzle, Andrea S. Winkler, Christine M. Budke, Hélène Carabin

**Affiliations:** 1 Department of Pathology and Microbiology, Faculty of Veterinary Medicine, Université de Montréal, Montréal, QC Canada; 2 Department of Social and Preventive Medicine, School of Public Health, Université de Montréal, Montréal, QC Canada; 3 Institute of Infectious Diseases and Tropical Medicine, Ludwig Maximilians University Hospital Munich, Ludwig Maximilians University Munich, Munich, Bavaria Germany; 4 Department of Neurology, Technical University of Munich Hospital, Technical University of Munich, Munich, Bavaria Germany; 5 Department of Community Medicine and Global Health, Institute of Health and Society, University of Oslo, Oslo, Norway; 6 Department of Veterinary Integrative Biosciences, College of Veterinary Medicine & Biomedical Sciences, Texas A&M University, College Station, TX USA; 7 Centre de Recherche en Santé Publique, Université de Montréal, Montréal, QC Canada; 8 Groupe de Recherche en Épidémiologie des Zoonoses et Santé Publique, Université de Montréal, Montréal, QC Canada

**Keywords:** Neurocysticercosis, *Taenia solium*, Epileptic seizures, Epilepsy, Systematic review, Bayesian meta-analysis, Meta-regression

## Abstract

**Background:**

Neurocysticercosis (NCC) is an infection of the central nervous system caused by the larval stage of *Taenia solium*. This systematic review (PROSPERO identifier: CRD42021266596) aimed to estimate the pooled proportion of NCC among people with epileptic seizures (PWES) between 1990 and 2023, and to assess whether this proportion changed between January 1990 to May 2008 and June 2008 to May 2023 across endemic regions.

**Methods:**

Twenty electronic databases were searched in May 2023 for records on NCC published between January 1, 1990, and May 9, 2023. Two reviewers independently screened all records. Original studies that reported NCC frequency among PWES, used neuroimaging, biopsy, or autopsy to diagnose NCC, and were not prone to high level of selection bias were included. Reviews, book-chapters and case series with fewer than 20 participants were excluded. Methodological quality of the included studies was assessed using the Joanna Briggs Institute checklist for prevalence studies. Bayesian models were used to estimate pooled NCC proportions and assess their changes by study periods and regions.

**Results:**

Among 16,202 screened records, 77 studies met the inclusion criteria. Thirty-five studies included PWES from both sexes, all age groups (children and adults), and used clear case definitions. The pooled proportion of NCC among PWES in these studies was 25.2% [95% Bayesian credible interval (*BCI*): 19.5%–32.2%], with fairly-high between-study heterogeneity (*τ* = 1.0; 95% *BCI:* 0.8–1.3). Studies without clear definitions for NCC or epileptic seizures reported considerably lower proportions, suggesting misclassification error. NCC proportion did not vary by sex or age. NCC proportion declined between June 2008 and May 2023, across Africa, the Americas and Southeast Asia.

**Conclusion:**

This study provides an updated estimate of NCC proportion among PWES between 1990 and 2023, with implications for burden estimation, and highlights its decline in endemic regions. However, substantial between-study heterogeneity indicates context-specific differences; therefore, the pooled estimates should be interpreted cautiously and not as universally generalizable. Further research should determine whether the observed decline in NCC proportion is real or reflects an apparent reduction resulting from changes in case definitions, study populations, neuroimaging technologies, or underlying causes of epileptic seizures.

**Graphical Abstract:**

**Figure:**
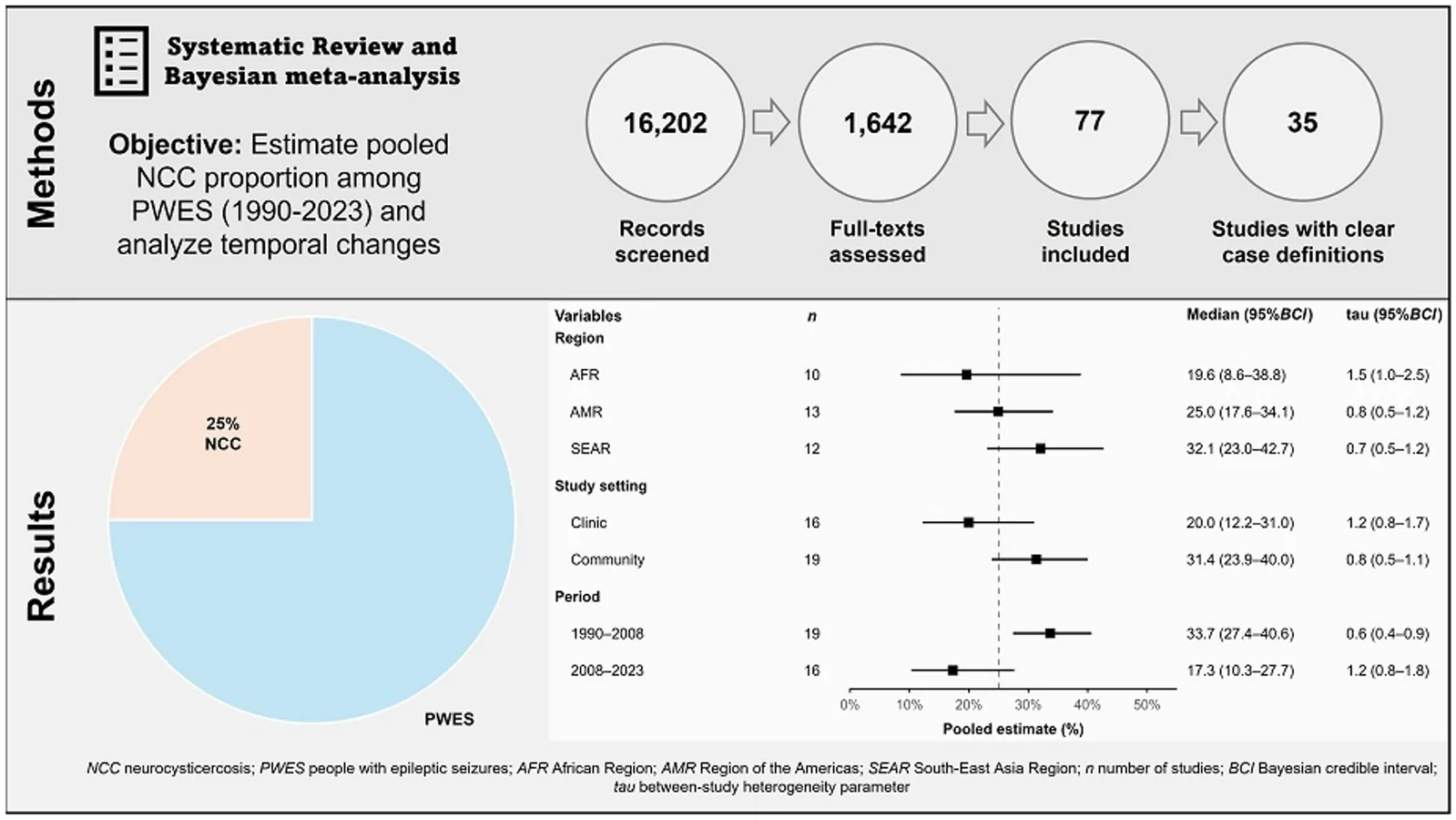

**Supplementary Information:**

The online version contains supplementary material available at https://doi.org/10.1186/s40249-026-01501-1.

## Background

Neurocysticercosis (NCC) is caused by infection of the central nervous system (CNS) with the larval form (*Cysticercus*) of *Taenia solium.* This helminthic zoonosis is considered a major cause of acquired epileptic seizures in humans worldwide [1, 2], with patients also presenting with several other neurological manifestations [3]. In 2010, *T. solium* was amongst the top five foodborne diseases in terms of disability-adjusted life years (DALYs) globally when only considering NCC-associated epilepsy [4]. Although NCC has been reported in low- and middle-income countries (LMICs) [5] and in high-income countries (HICs) [2, 6], its frequency in the general population remains unknown, largely due to the lack of easily accessible and inexpensive diagnostic tools. The recognized diagnostic criteria for NCC are either brain imaging [e.g., computed tomography (CT) or magnetic resonance imaging (MRI)], biopsy or autopsy [7–13]. Brain imaging is often unavailable or poorly accessible in countries where NCC is endemic [14, 15], and brain biopsies or autopsies are uncommon. Limited use of recognized diagnostic criteria makes it challenging to accurately measure the frequency and burden of NCC. As a result, it is difficult to evaluate risk factors for acquiring NCC and the effectiveness of control strategies for NCC globally. This also means that the global burden of this zoonosis must be measured indirectly, primarily using research data or clinical data from specific populations rather than surveillance data.

In 2010, a systematic review with meta-analysis of the literature published between January 1, 1990 and June 1, 2008 estimated the pooled proportion of NCC among people with epilepsy (PWE) in endemic areas at 29.0% [95% confidence interval (95% *CI*) 22.9%–35.5%] [16]. This estimate was used by the World Health Organization’s Foodborne Disease Burden Epidemiology Reference Group for 2007–2015 (WHO-FERG) to estimate the global burden of NCC [4, 17]. However, this estimate of the proportion of NCC among PWE used data from studies conducted more than 15 years ago, and the situation may have changed in the interim for two main reasons. First, improvements in sanitation and implementation of *T. solium* control programs may have resulted in a reduction in the proportion of NCC among PWE. Second, improved access to brain imaging facilities and improved quality of the imaging equipment could have resulted in an increase or decrease in this proportion. It is also possible that such tendencies varied regionally. The primary objective of this study was to estimate the pooled proportion of NCC among people with epileptic seizures (PWES) reported in the literature between January 1, 1990 and May 9, 2023. The secondary objective was to assess if the proportion of NCC among PWES changed between January 1990 to May 2008 and June 2008 to May 2023 and if this change varied according to region.

## Methods

### Protocol and registration

The protocol for this systematic review and meta-analysis was registered in PROSPERO (Reg. No: CRD42021266596) on August 7, 2021, and later modified based on input from the WHO-FERG [18, 19]. Notably, although the registered protocol outlined a broader scope encompassing both people with epilepsy and those with headaches, the present manuscript focuses exclusively on individuals with epileptic seizures. The findings related to headaches and NCC among other study populations are presented in a separate manuscript. The systematic review is reported following the Preferred Reporting Items for Systematic Reviews and Meta-Analyses (PRISMA) guidelines [20] (the complete checklist is available in Supplementary Table S1).

### Information sources

Twenty stable databases were searched based on the strategy adopted by Ndimubanzi et al. (2010) and following the recommendations from the WHO-FERG [16, 21]. These databases include—Africabib, CAB Direct, Embase, Global Health, Google Scholar, GrayGuide, Hispanic American Periodicals Index, INASP journal online project, Latin American Open Archives Portal, Oaister, PubMed (MEDLINE), SciELO-Scientific Electronic Library Online, Scopus, Web of Science, WHO Library, WHO-African Index Medicus, WHO-Index Medicus for Eastern Mediterranean Region, WHO-Latin America and the Caribbean Literature on Health Sciences, WHO-Index Medicus for South-East Asia Region, and WHO-The Western Pacific Region Index Medicus (links for these databases are provided in Supplementary Table S2). The search was conducted between May 9 and May 29, 2023. A snowballing strategy was adopted to identify additional publications from the reference lists of review articles on NCC and from articles included in the full-text review.

### Search strategy

The general search parameters are presented in Table 1, while database-specific search algorithms are provided in Supplementary Table S2. For example, the search in PubMed used the following algorithm:

**Table 1:** Search strategy used to find articles reporting the frequency of neurocysticercosis

Trait	Description
Keywords with Boolean operators^a^	“Neurocysticercosis” OR “Cysticercosis” OR “*Taenia solium*” OR “Taeniasis” OR “Taeniosis”
Year range	1990–2023
Level of search for keywords	Full text

^a^If there was no option to use Boolean operators, the search was conducted using one keyword (e.g., “Taeniasis”) at a time and then adjusted by combining all lists of literature and removing duplicates using EndNote version 20.4.1 (Clarivate, Philadelphia, USA) software

(“Neurocysticercosis/epidemiology” [MeSH] OR “Neurocysticercosis” OR “Cysticercosis” OR “Taenia solium” OR “Taeniasis” OR “Taeniosis”), limited to studies published between 1990 and 2023.

The search strategy was adopted to capture a wide range of papers recognising that diverse terminologies may be used by researchers to denote NCC, namely cerebral cysticercosis, *T. solium* cyst in brain, etc. In the case of bilingual (e.g., Vietnam Journals Online) or multilingual (e.g., SciELO) databases, additional searches were conducted using translated keywords. The extended inclusion period (from 1990 to 2023) was intended to maximise the retrieval of all relevant evidence, including both earlier and more recent studies, as well as those that may not have been captured in the previous meta-analysis [16] due to differences in database coverage, indexing, or search terminology.

### Study selection process

The systematic review included three phases—(i) Phase 1: Title and abstract screening; (ii) Phase 2: Full text review of articles included following Phase 1; and (iii) Phase 3: Quality assessment and data extraction of included articles following Phase 2. In Phase 1, the titles and abstracts were screened using Covidence (Veritas Health Innovation Ltd, Melbourne, Australia) online software by two independent reviewers (for each title and abstract, MSJ acted as first reviewer, while either OF or KD acted as the second reviewer) [22]. Any disagreement between the reviewers was resolved upon discussion. If an agreement could not be reached, a senior researcher (HC or CB) was consulted until a resolution was found. In Phase 2, all articles deemed eligible following Phase 1 were read in full by two independent reviewers (for each article, MSJ acted as first reviewer, while either OF or MH acted as the second reviewer) to determine whether to include or exclude them from further analysis based on the eligibility criteria (see the section “Eligibility criteria”). Any disagreement between the two reviewers was discussed in a face-to-face meeting. A senior researcher (HC or CB) was consulted to resolve any remaining disagreements following that meeting. Articles written in a language other than English were reviewed by a reviewer proficient in that language, along with a second reviewer who used online translation tools (DeepL or Google Translate) to translate the document into English. A description of Phase 3 is given later (see the section “Quality assessment and data collection process”).

### Operational definition of terms used in this study

#### Epileptic seizure

Diagnosis of epileptic seizures can be based on the definition from the International League Against Epilepsy (ILAE), other sources (e.g., any previous study) or expert opinion. The formal definition from the ILAE is, “a transient occurrence of signs and/or symptoms due to abnormal excessive or synchronous neuronal activity in the brain” [23]. Since epileptic seizures are difficult to define operationally [23], we considered any type of seizure or epilepsy described by the author as an “epileptic seizure.” Accordingly, the term “people with epileptic seizures (PWES)” was used as an umbrella term encompassing both people with epilepsy (see definition of epilepsy below) and people with epileptic seizures other than epilepsy. For studies conducted in a specialized clinic, secondary hospital or tertiary hospital, it was assumed that the diagnosis of epileptic seizures was done by an expert in neurology. The same assumption was made for studies where the diagnosis of epileptic seizures was based on the International Classification of Diseases (ICD) code reported in medical charts or administrative databases. Studies where the diagnosis of epileptic seizures was based solely on expert opinion, without citing a specific definition or reference, were considered as not providing a formal definition for epileptic seizures.

#### Epilepsy

The formal definition of epilepsy has been refined several times since 1981 [23–28]. Specifically, ILAE and the International Bureau for Epilepsy (IBE) defined epilepsy in 2005, as “a disorder of the brain characterized by an enduring predisposition to generate epileptic seizures and by the neurobiologic, cognitive, psychological, and social consequences of this condition” [23]. The definition of epilepsy requires the occurrence of at least one epileptic seizure [23]. In 2014, the ILAE proposed a practical definition that considered epilepsy a disease of the brain defined by any of the following conditions: (1) at least two unprovoked (or reflex) seizures occurring more than 24 h apart; (2) one unprovoked (or reflex) seizure and a probability of further seizures similar to the general recurrence risk (at least 60%) after two unprovoked seizures, occurring over the next 10 years; (3) diagnosis of an epilepsy syndrome [28]. We attributed a diagnosis of “epilepsy” to a study population if the diagnosis was assigned based on any version of the ILAE definition, on a definition reported in a previous research article or on expert opinion. Accordingly, the term “people with epilepsy (PWE)” refers to individuals whose clinical manifestations met one of the diagnostic criteria for epilepsy described above.

#### Epileptic seizures other than epilepsy

We classified study populations as having epileptic seizures other than epilepsy when the reported clinical manifestations were described as any form of seizure (e.g., single seizures, first-time seizures) that did not meet the ILAE definition of epilepsy [23–28] or when there was insufficient information to determine whether the ILAE criteria for epilepsy were fulfilled.

#### NCC

NCC cases were defined as those confirmed using brain imaging (e.g., CT-scan or MRI), biopsy or autopsy. The interpretation of brain imaging results was based on formal criteria described in previous research (e.g., [7–13]) or expert opinion. For studies conducted in a specialty clinic, secondary hospital, or tertiary hospital, and when not otherwise mentioned, it was assumed that the interpretation of brain images was done by a radiologist or an expert in neurology or neuroimaging. The same assumption was made for studies where the diagnosis of NCC was based on ICD codes reported in medical charts or administrative databases. Studies where the diagnosis of NCC was based solely on expert opinion without citing a specific definition or reference, were classified as being *without* a formal definition for NCC.

#### Studies without or with formal definitions

Studies where the diagnosis of epileptic seizures, epilepsy or NCC was solely based on expert opinion were deemed as “*without* formal definition.” Studies were categorized as “*with* formal definitions” when both NCC and epilepsy or epileptic seizures were diagnosed based on recognized definitions as described above or were referring to clear definitions reported in previous research.

#### Study periods (period A and B)

The articles included in the meta-analysis were categorized into two time periods: period A (January 1, 1990 to May 31, 2008) and period B (June 1, 2008 to May 9, 2023). If all or most of the data collection took place before June 2008, the article was classified as period A, while it was classified as period B if all or most of the data collection took place from June 2008 onward. This categorization allowed us to assess if the proportion of NCC among PWES changed since the meta-analysis published in 2010 [16], which only included studies conducted during period A.

#### Regions

The study region was classified using the WHO regions–African Region (AFR), Region of the Americas (AMR), South-East Asian Region (SEAR), European Region (EUR), Eastern-Mediterranean Region (EMR) and Western Pacific Region (WPR) [29].

### Eligibility criteria

Any publication with original data from a cross-sectional, case-control, cohort, case series/report (with at least 20 participants) or hybrid study which measured the frequency of NCC among PWES (i.e., people with epilepsy and people with epileptic seizures other than epilepsy), was considered eligible for this review. In Phase 1, articles for which titles and abstracts met at least one of the following criteria were excluded—(i) the reported data were related to the wrong agent, such as *T. saginata*, or there were no data on *T. solium*, (ii) only animal data were reported, (iii) only the clinical features of NCC patients were reported without data on the frequency of NCC among PWES, (iv) the study was a case series or case report with less than 20 cases, (v) any review article or book chapter (if authors described the article as a “review” or “chapter” in the abstract) without original data, and (vi) editorials or letters to the editors (if the authors described the article as an “editorial” or “letter” in the abstract). In Phase 2, the criteria used in Phase 1 were applied in addition to three additional exclusion criteria—(i) no neuroimaging (CT-scan or MRI), brain biopsy or autopsy used for the diagnosis of NCC; (ii) unable to confirm how the epileptic seizures or epilepsy were diagnosed; and (iii) the sampling strategy was prone to misrepresentation of the target population (e.g., the study population over-represented people living with a person with taeniasis). In cases of multiple articles reporting the same data, one article was included for further evaluation.

### Quality assessment and data collection process

In Phase 3, data from studies included following the review in Phase 2 were extracted using a modified Microsoft Access tool originally developed by Ndimubanzi et al. (2010) [16]. Modifications included the addition of the Joanna Briggs Institute (JBI) critical appraisal checklist for studies reporting prevalence data (the checklist is given in Supplementary Appendix S1) [30]. In this review, the JBI checklist was used to evaluate the methodological quality of included studies and to identify missing information related to the selection of study populations, but the results were not used as exclusion criteria. The modified version of the Microsoft Access tool includes 85 variables listed in Supplementary Table S3. All data were independently extracted by two reviewers (MSJ extracted data from all studies, with either OF or MH serving as the second reviewer). Disagreements were resolved through discussion among all reviewers. When information was missing from a paper, the corresponding author of the study was contacted at least once via email.

### Data items

The following data were extracted in Phase 3: study characteristics (e.g., study design, study setting, geographic location, period and duration), sampling strategy, participants’ demographic characteristics (e.g., age, sex), definition of epileptic seizures or epilepsy, definition or diagnostic criteria for NCC, type of brain imaging used, number (e.g., single, 2–10, > 10) and the type (e.g., active, degenerative, calcified) of brain lesions, and number of NCC cases according to the characteristics described above, when available.

### Summary measures and synthesis of the results

The geographical location of eligible studies was depicted on a world map using Microsoft Excel 365 version 2504 (Microsoft Corporation, Redmond, United States). The flowcharts presented in this article were created using the online software draw.io (JGraph Ltd., London, United Kingdom). All quantitative data analyses were performed in the statistical computing ecosystem of RStudio version 2024.04.2 (Posit Software, Boston, United States) and R version 4.4.2 (R Foundation for Statistical Computing, Vienna, Austria). The summary measure of interest was the proportion of NCC among PWES. Study-specific proportions of NCC among PWES were estimated using the *rstan* R-package [31], which allowed Bayesian logit-binomial modeling of each study’s binomial data. To combine data from multiple studies, Bayesian random effects logistic models (also known as Bayesian multilevel/hierarchical models) were fitted using the *brm()* function from the *brms* R-package [32, 33]. Both packages allow users to implement the Bayesian models in Stan’s probabilistic language from the R interface. Stan uses the No-U-Turn Sampler (NUTS), an extension of the Hamiltonian Monte Carlo (HMC) algorithm, for sampling. Four Markov chains of 10,000 iterations each (including 5000 burn-in iterations) were run for each model. The R-hat (Gelman-Rubin statistics) value of the model along with model diagnostic plots (e.g., posterior predictive density plot, trace plot, and posterior predictive check plot), were generated to assess the convergence of the model [34]. Convergence was reevaluated by doubling the number of iterations (e.g., 20,000 for each chain) [35]. Posterior summaries (median and 95% Bayesian credible intervals [*BCI*]) from converged models were transformed from the logit to the probability scale using the *plogis()* function in base-R and multiplied by 100 to express the results as percentages. Between-study heterogeneity was assessed by directly interpreting the estimate for the group-level hyperparameter (*τ* = estimate for standard deviation of the intercept). Values of *τ* from 0.1 to 0.5 indicate “reasonable” heterogeneity, those between 0.5 and 1.0 indicate “fairly high” heterogeneity and those above 1.0 indicate “fairly extreme” heterogeneity [33, 36, 37]. Forest plots were generated using the *ggplot2* package in R [38]. For community-based cross-sectional studies *with* formal definitions, we also estimated the prevalence of NCC-associated epilepsy in the community. This estimate is the product of the prevalence of epilepsy in a community and the proportion of NCC among PWE in the same community.

### Subgroup meta-analyses and meta-regressions

Several subgroup meta-analyses were conducted. The study selection process for the subgroup analyses and meta-regressions is illustrated in Supplementary Fig S1. The first subgroup analysis estimated the proportion of NCC among PWES separately for studies *with* and *without* formal definitions as described above using all relevant studies. The results of this first subgroup analysis led us to conduct the remaining subgroup analyses and meta-regressions only among studies *with* formal definitions. These included subgroup meta-analyses according to the study period (A and B), type of manifestations present in the study population (people with epilepsy and people with epileptic seizures other than epilepsy), study setting (clinic/hospital and community), and region (AFR, AMR, and SEAR). Additionally, stratified estimates of the proportion of NCC among PWES by age group (children and adults) and sex (male and female) were calculated using data from studies for which these subgroup data were available. To assess if the proportion of NCC among PWES changed after June 2008, several meta-regressions using Bayesian mixed-effect logistic regression models, including the study period and several potential confounders or effect modifiers were run. Prevalence proportion ratios (*PR*s) and their 95% *BCI*s were derived from the posterior samples by converting the log-odds estimates to probabilities followed by the computation of the ratio between the two categories of each covariate (e.g., period B vs period A).

### Prior selection and sensitivity analysis

For the meta-analysis models, very weakly informative *logistic* and *Half-Cauchy* prior distributions were assumed for the proportion (*P*) and between-study heterogeneity (*τ*) parameters, respectively.

P∼logistic(0,1)

$$\tau \sim HalfCauchy\left(0, 0.5\right)$$

For the meta-regression models, an additional normal prior distribution with a mean of zero was assumed for each beta coefficient (*β*).

$$\beta \sim Normal(0, 1)$$

All models were re-run using other weakly informative priors to assess the robustness of the data [33, 39]. However, only the sensitivity analysis results for the meta-analysis, including studies *with* and *without* a formal definition are presented in this manuscript.

## Results

### Study selection

**Fig. 1:**
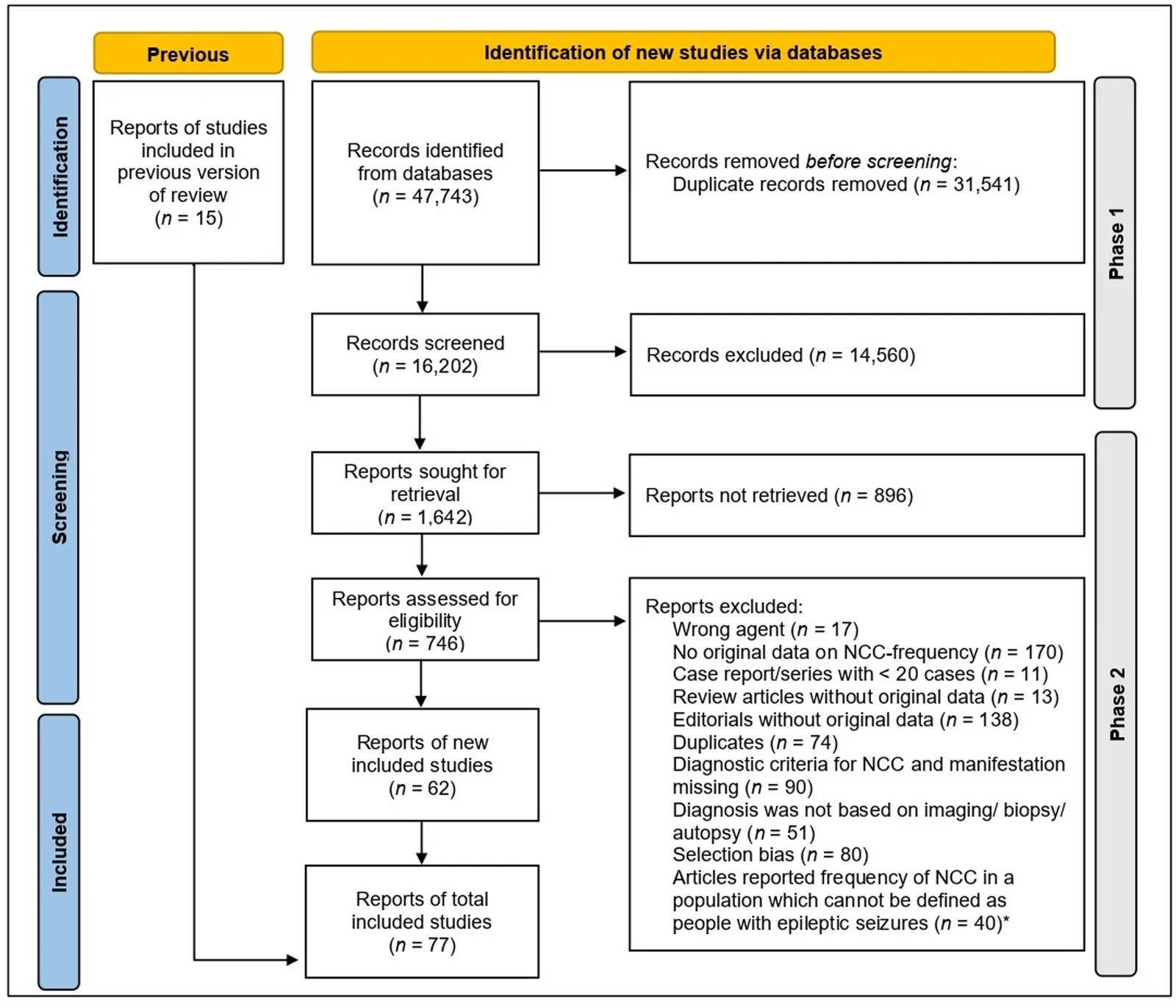
Preferred Reporting Items for Systematic Reviews and Meta-Analyses (PRISMA) flowchart of the study selection process. * “People with epileptic seizures (PWES)” are defined in the section “Operational definition of terms used in this study” in the main manuscript. Studies involving people who do not meet the operational definition of PWES were excluded from the present analysis and are reported in a separate manuscript. *NCC* neurocysticercosis; *n* number of records/studies

Figure 1 depicts the flow chart of the article selection process. A total of 47,743 records were uploaded to Covidence. Of these, 31,541 duplicates were identified and removed automatically by Covidence or through manual inspection, resulting in 16,202 records available for Phase 1 screening. A total of 1642 articles were deemed eligible for full-text review, of which 102 were selected for data extraction. Sixty-two (61 peer-reviewed articles and one thesis) reported data on the frequency of NCC among people who underwent brain imaging after presenting with epileptic seizures. Fifteen additional articles with similar data were included from Ndimubanzi’s review [16]. This resulted in a total of 77 articles (38 from period A and 39 from period B) included in the current review.

### Study characteristics

A detailed description of each study is given in Supplementary Table S4. Included studies were conducted across 23 countries from the AFR, AMR, SEAR, EUR and EMR regions (see the geographical distribution of the included studies in Supplementary Fig S2). The majority of included articles were published in English (*n* = 67), followed by Spanish (*n* = 4), Portuguese (*n* = 4), and French (*n* = 2). Fifty-two studies were clinic/hospital-based and 25 were community-based. Forty-five studies included both children (< 18 years old) and adults (≥ 18 years old), whilst 17 included only children and 11 included only adults. It was impossible to determine the age group of the study population in four studies (see Supplementary Fig S1). Sex-specific data were available in 10 studies.

### Results of individual studies

Results of individual studies are provided in Supplementary Table S5. The study-specific estimates of the NCC proportion among PWES varied from 1.7% (95% *BCI:* 0.3%–5.6%) to 97.7% (95% *BCI:* 88.2%–99.9%) depending on the study population (see Supplementary Table S5). Data from 59 studies were included in the meta-analysis or in the subgroup meta-analysis comparing studies *with* or *without* formal definitions. For one study conducted in Tanzania, the unadjusted NCC-proportion estimates are presented in Supplementary Table S5, but only the results weighted for verification bias were included in the meta-analyses. The weighted estimate of the proportion with NCC was 35.0% (95% *BCI:* 30.0%–39.0%) among PWES [40]. Data from six studies and the crude results from the study described above [40] were not included in the meta-analysis because they were conducted among very specific study populations [41–46].

In the Tanzanian study [40], the authors reported that 77.0% (95% *BCI:* 65.4%–86.6%) and 26.8% (95% *BCI:* 13.7%–43.9%) of PWES who tested positive and negative on a point-of-care (POC) test to detect taeniosis and (neuro)cysticercosis antibodies had NCC lesions in their brain, respectively [40]. In a cross-sectional study conducted in 13 villages located in Mbozi district, Tanzania, all 28 participants with a history of seizures who tested positive for *T. solium* cysticerci antigen (B158/B60) were diagnosed with NCC on CT-scan examination [45]. In a study conducted among international children adopted in Italy, the proportion of NCC was 79.9% (95% *BCI:* 47.2%–96.8%) [44]. The remaining four studies were conducted in India [41–43, 46]. A case series of 150 cases reported an NCC proportion of 2.4% (95% *BCI:* 0.7%–5.7%) among children (aged one to 12 years) with partial motor seizures who attended the department of paediatrics and radiology at the Jawaharlal Institute of Postgraduate Medical Education and Research, Pondicherry [46]. This study excluded children who had developmental delay, febrile seizures or when partial seizures were associated with acute intracranial infection or any other acute neurological insult [46]. In Northern India, Singhi et al. reported an NCC proportion of 13.5% (95% *BCI:* 7.6%–20.8%) among children (aged three months to 12 years) with onset of partial seizures in “the past month” who had both a CT-scan and electroencephalography. This study excluded patients with a history of neurological insult or persistent neurological signs [43]. Another hospital-based study from South India identified a higher proportion of NCC (40.0%; 95% *BCI:* 32.2%–48.0%) among children (aged one month to 18 years) who had brain imaging after experiencing one or more focal seizures, excluding those with generalized tonic–clonic or atonic seizures [42]. Sil et al. (2012) reported a similar proportion of NCC among PWES from all ages attending a tertiary care hospital in Eastern India [41]. In that study, the proportion of NCC was 40.6% (95% *BCI:* 30.1%–51.8%) among individuals with idiopathic epilepsy or seizures due to a granuloma who had been receiving treatment for at least two months. However, this study excluded patients who had cerebral palsy or who were hospitalized [41].

### Pooled proportion of NCC among people with epileptic seizures of all ages

**Fig. 2:**
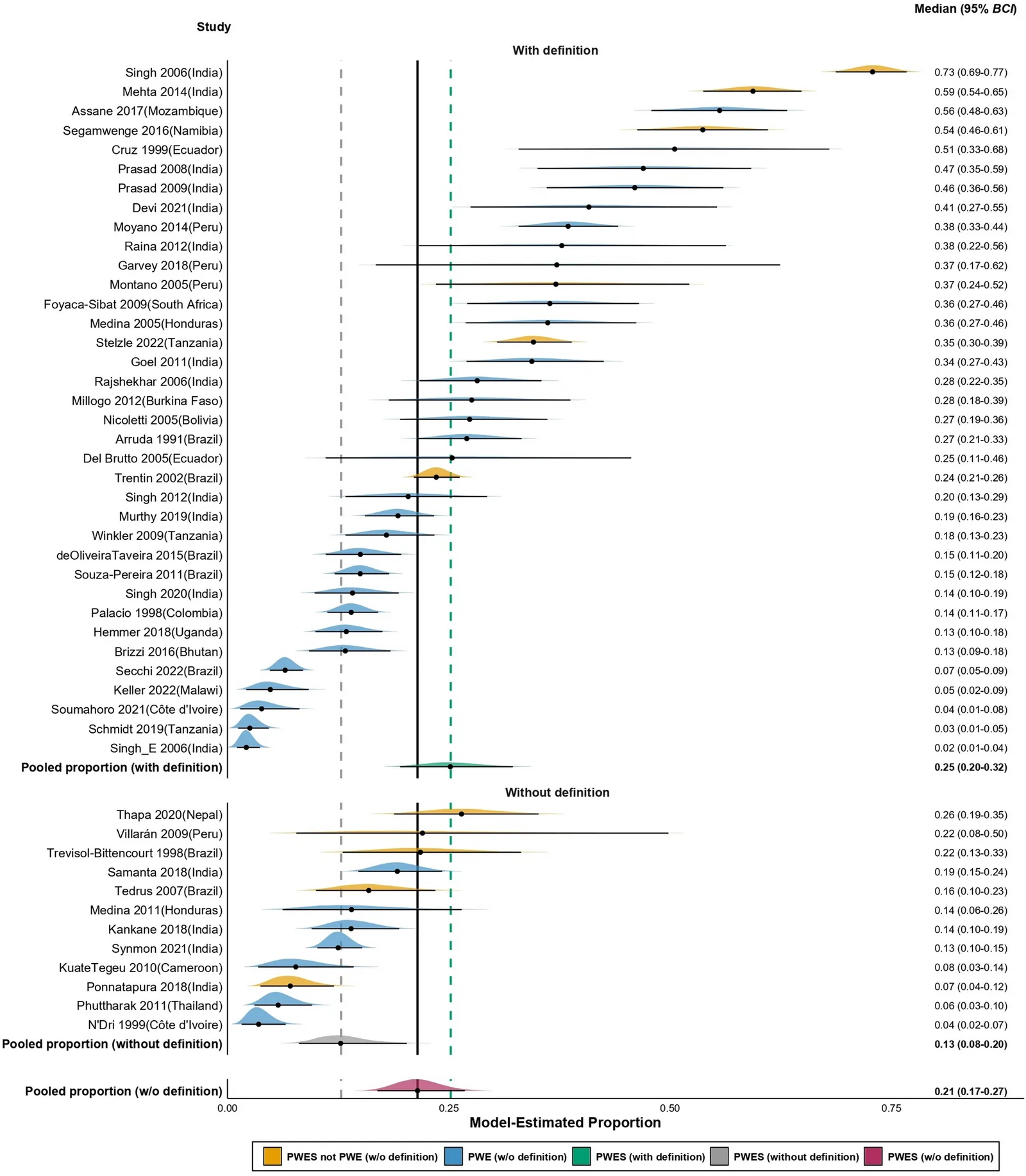
Forest plot showing density graphs of the posterior distributions for all included studies and pooled estimates of NCC among PWES. The black vertical solid line represents the overall pooled proportion of NCC among PWES estimated from studies *with* or *without* definitions. The grey dashed line represents the pooled proportion derived from studies *without* definitions [PWES (without definition)], whereas the green dashed vertical line represents the pooled proportion from studies *with* definitions [PWES (with definition)]. The blue shapes depict the posterior distributions of the study-specific proportions of NCC among people with epilepsy in studies *with* or *without* definitions [PWE (w/o definition)], while the orange shapes represent the posterior distributions of study-specific proportions among people with epileptic seizures other than epilepsy in studies *with* or *without* definitions [PWES not PWE (w/o definition)]. The grey shapes represent the posterior distribution of the pooled proportions from studies *without* definitions and and the green shapes depicts those from studies *with* definitions. The maroon shape shows the posterior distribution of the overall pooled proportions of NCC among PWES from both study types combined [PWES (w/o definition)]. Black dots indicate the median estimates, and the accompanying black vertical lines represent their corresponding 95% Bayesian credible intervals. *PWES* people with epileptic seizures; *BCI* Bayesian credible interval; *w/o* with or without

The pooled proportion of NCC among PWES of all ages was estimated based on 47 studies (study selection tree is illustrated in Supplementary Fig S1), which reported the frequency of NCC among PWES across all age groups or the age group of the study population was not specified. For the latter, we assumed the study populations included both children and adults. Twenty-six studies were from period A and 21 from period B. Included studies represented 10,371 PWES of whom 2,358 were diagnosed with NCC. The pooled proportion of NCC among PWES was 21.5% (95% *BCI:* 16.9%–26.8%) for both study periods with “fairly high” between-study heterogeneity (*τ* = 1.0; 95% *BCI:* 0.8–1.3) (see Fig. 2). This estimate includes studies *with* and *without* formal definitions for NCC and epileptic seizures and/or epilepsy and is prone to misclassification bias.

### Impact of the use of formal definitions for NCC and epileptic seizures or epilepsy on the pooled proportion of NCC among PWES of all ages

Figure 2 depicts the forest plots for 47 studies *with* (*n* = 35) and *without* (*n* = 12) definitions, together with pooled estimates for each subgroup and overall. In studies *with* formal definitions, the pooled proportion of NCC among PWES of all ages was 25.2% (95% *BCI:* 19.5%–32.2%), while it was considerably lower at 12.8% (95% *BCI:* 8.1%–20.3%) for studies *without* formal definitions.

### Subgroup meta-analyses among studies *with* definitions

The results of all subgroup meta-analyses, using studies *with* definitions, are presented in Supplementary Fig S3. Using the 35 studies *with* formal definitions, the proportion of NCC among PWES was 33.7% (95% *BCI:* 27.3%–40.7%) for period A (*n* = 19) and 17.3% (95% *BCI:* 10.2%–27.8%) for period B (*n* = 16). Community-based studies (*n* = 19) showed an estimate of 31.4% (95% *BCI:* 23.9%–40.3%) compared to 20.0% (95% *BCI:* 12.3%–31.2%) in clinic/hospital-based studies (*n* = 16).

The posterior distributions were similar across regions, with an NCC proportion of 32.1% (95% *BCI:* 23.0%–43.0%) in SEAR (*n* = 12), 25.0% (95% *BCI:* 17.7%–34.5%) in AMR (*n* = 13) and 19.6% (95% *BCI:* 8.5%–40.3%) in AFR (*n* = 10). The NCC proportions were extremely heterogenous across studies from AFR (*τ* = 1.5; 95% *BCI:* 1.0–2.5), while fairly high heterogeneity was observed in AMR (*τ* = 0.8; 95% *BCI:* 0.5–1.2) and SEAR (*τ* = 0.7; 95% *BCI:* 0.5–1.2). The proportion of NCC was 20.8% (95% *BCI:* 14.8%–28.5%) among PWE (*n* = 30) and 47.3% (95% *BCI:* 30.5%–65.1%) among people with epileptic seizures other than epilepsy (*n* = 6).

**Fig. 3:**
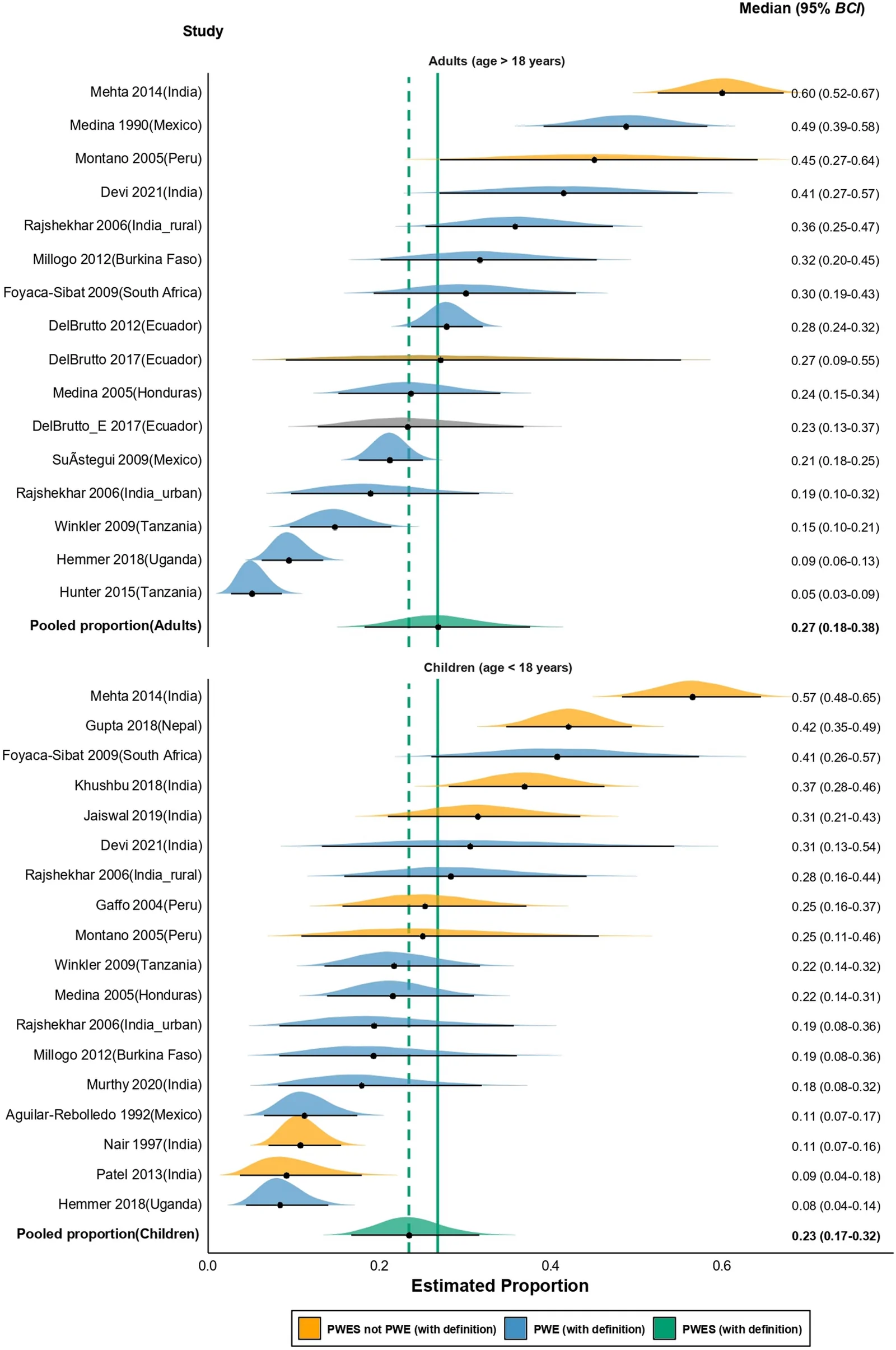
Forest plot showing the density graphs of age group-stratified posterior distributions of NCC proportion for individual studies and pooled estimate for each stratum. The green vertical solid line represents the pooled proportion of NCC among adults with epileptic seizures, and the green dotted vertical line represents the pooled proportion among children with epileptic seizures. The blue shapes depict the posterior distributions for the study-specific proportions of NCC among people with epilepsy in studies *with* or *without* definitions [PWE (w/o definition)], while the orange shapes represent the posterior distributions of study-specific proportions among people with epileptic seizures other than epilepsy in studies *with* or *without* definitions [PWES not PWE (w/o definition)]. The green shapes illustrate the posterior distribution of the overall pooled proportions for adults in the upper panel and for children in the lower panel. Black dots indicate the median estimates, and the accompanying black vertical lines represent their corresponding 95% Bayesian credible intervals. *PWE* people with epilepsy; *PWES* people with epileptic seizures; *BCI* Bayesian credible interval

Only eight studies *with* definitions reported sex-specific data (see Supplementary Fig S1 and Table S6). The estimated proportion of NCC among PWES was similar in males (23.2%; 95% *BCI:* 9.0%–49.4%; *n* = 8) and females (24.3%; 95% *BCI:* 9.8%–50.6%; *n* = 8). Subgroup analyses for age were conducted on studies providing information on both children and adults (*n* = 9) as well as on studies (*with* definitions) conducted among children only (*n* = 8) or adults only (*n* = 5) (see Supplementary Fig S1 and Table S6). The proportion of NCC among children and adults with epileptic seizures was 23.4% (95% *BCI:* 16.7%–31.7%; *n* = 17) and 26.8% (95% *BCI:* 18.1%–38.0%; *n* = 14), respectively. The forest plot representing the Bayesian meta-analyses results for age group strata is presented in Fig. 3.

### Effect of the study period on the proportion of NCC among PWES

Meta-regression, including 35 studies *with* definitions showed that the proportion of NCC among PWES declined in period B compared with period A. The *PR* for period B was 0.5 (95% *BCI:* 0.3–0.8). The *PR* was unchanged when the regression model was adjusted for potential confounders (e.g., region, imaging technology used or study settings). These results are presented in the supplemental materials (see Supplementary Appendix S2). The effect of the period was not modified by region, with the magnitude of the reduction in NCC proportion in period B similar in all three regions (see Supplementary Appendix S2).

### Prevalence of NCC-associated epilepsy in community-based studies

We identified 17 community-based studies where it was possible to estimate the prevalence of NCC-associated epilepsy among a sample of the general population. All studies provided definitions for NCC and epilepsy and included people from all age groups (children and adults) and both sexes (male and female). The prevalence of NCC-associated epilepsy ranged from 0.03% (95% *BCI:* 0.01%–0.06%) in a study from Malawi to 5% (95% *BCI:* 4%–6%) in a study from Mozambique (see Supplementary Table S7). The prevalence of epilepsy ranged from 0.3% (95% *BCI:* 0.3%–0.4%) to 8% (95% *BCI:* 6.9%–9.3%), while the prevalence of NCC among PWE varied from 3.5% (95% *BCI:* 1.2%–7.7%) to 53.7% (95% *BCI:* 35.3%–71.2%).

### Sensitivity analysis

The impact of using different sets of priors was evaluated using all studies (i.e., those *with* and *without* formal definitions) and the results are given in the supplemental materials (see Supplementary Table S8). No substantial changes in NCC proportion estimates were observed by changing the prior for both the *P* and *τ* parameters. The median proportion estimates varied from 21.1% (95% *BCI:* 16.4%–26.6%) to 21.7% (95% *BCI:* 16.9%–27.4%), while the heterogeneity estimates were consistently around 1.0 (95% *BCI:* 0.8–1.3).

## Discussion

Our meta-analysis provides a comprehensive and updated estimate of the proportion of NCC among PWES in NCC-endemic WHO regions between 1990 and 2023. We found that approximately one quarter of PWES had NCC lesions on imaging in endemic areas, which is slightly lower than what had been reported for the period between 1990 and 2008 by Ndimubanzi et al. (2010). Our revised estimate for that period was higher but with overlapping credible intervals [16]. The difference stems from the addition of eight studies, six of which were published after Ndimubanzi et al. (2010) conducted their literature search in 2008. Differences in database coverage and article search strategies also contributed to the discrepancy. We searched relatively stable databases using a homogenous search algorithm to capture as many peer-reviewed and gray literature publications as possible. Our broad search is reflected in the much larger number of citations initially identified.

An important finding of our study is the impact of using formal definitions for epilepsy, epileptic seizures and NCC on the results. Our results show that studies *with* definitions estimated the proportion of NCC among PWES to be nearly twice that of studies *without* definitions. This difference can be explained by two factors. First, in studies *without* formal definitions, case ascertainment for both NCC and epileptic seizures relies heavily on individuals’ clinical judgment [47], which can introduce systematic misclassification bias. NCC diagnosis requires the interpretation of neuroimaging findings [13], and diagnostic uncertainty can arise because some neuroimaging findings in NCC patients are nonspecific (e.g., a single ring-enhancing lesion may also occur in tuberculoma [48]) [7]. Conversely, epileptic seizures are often diagnosed based on direct observation or self-reported symptoms [47], without systematic classification, resulting in the inadvertent inclusion of non-epileptic or ambiguous cases. Both processes would systematically dilute the proportion of NCC among PWES. Second, studies that applied formal definitions for both NCC and epileptic seizures systematically assessed which seizures were attributable to NCC using structured evaluation frameworks. Without such criteria [7–13], seizures may be classified as idiopathic or of unknown etiology, resulting in undercounting of NCC-associated cases.

There was also heterogeneity among studies *with* definitions, likely reflecting differences in diagnostic criteria, study period, and context. First, no universally accepted gold standard exists for diagnosing both NCC and epileptic seizures or epilepsy. Formal diagnostic standards for NCC were introduced in 1996 and revised in 2001 and 2017 [7, 8, 11–13]. Besides the above-mentioned standards, some included studies used criteria for the diagnosis of NCC dating from the 1980s (e.g., [49] and [50]). The definitions of epileptic seizures and epilepsy have also evolved over the last six decades [24–27, 51]. These changes likely introduced misclassification bias across studies. Studies using older diagnostic criteria for NCC may have overestimated its prevalence by including probable or uncertain cases. Conversely, studies using the most recent criteria may have underestimated NCC prevalence by excluding suspected cases that would have been considered NCC under earlier definitions. In addition, differences in diagnostic technologies across studies could have further influenced ascertainment of NCC and/or epileptic seizures cases.

Confirmation of NCC cases requires brain imaging, such as CT-scan or MRI [52], while an electroencephalogram (EEG) remains important for differential diagnosis of epileptic and non-epileptic seizures [53]. Most included studies used CT-scans, which perform well at detecting calcified lesions, but are less sensitive for cystic-stages and smaller NCC lesions [8]. In contrast, studies relying solely on MRI may have missed NCC with calcified lesions. EEG was seldom used to distinguish epileptic from non-epileptic seizures. The combined use of EEG, CT-scan and MRI, along with interpretation of the imaging results by multiple experts, might improve the accuracy of NCC and epileptic seizures case ascertainment, but such studies are challenging to conduct in NCC-endemic regions of LMICs because brain imaging is often unavailable or poorly accessible [14, 15]. However, the situation may have changed in some LMICs over time [54–56].

The second source of heterogeneity among studies was time (measured by study period). We observed a lower proportion of NCC among PWES in period B than in period A. This decline may be real or an apparent, but spurious, reduction. A real decline may be attributable to cysticercosis control measures, including improved sanitation [57], health education [57], mass drug administration [58] and pig management, implemented across endemic countries over the past 15 years [59, 60]. An apparent, but spurious, reduction may be the result of temporal variations in the precision, availability, and accessibility of diagnostic technologies [54–56, 61, 62], which could have introduced measurement bias. Improvements in brain imaging technologies, together with the application of updated diagnostic criteria for NCC [8, 13], have likely increased diagnostic accuracy and reduced false positive cases. However, our analysis suggests that improved access to better equipment is unlikely to be the cause of the observed reduction with time since we observed similar proportions of NCC and levels of reduction through time across regions. If access to technology was the cause of the observed reduction, we should have observed regional variations.

Another reason for an apparent reduction could be a change in the underlying causes of epileptic seizures through time. Epileptic seizures may result from infectious diseases (e.g., meningitis and encephalitis due to bacteria, viruses or parasites) and non-infectious brain conditions (e.g., stroke, head trauma, and cancer), fever, and genetic or metabolic disorders (e.g., diabetes mellitus) [63, 64]. Although the global burden of epileptic seizures increased between 1990 and 2021 [65], the declining proportion of NCC among PWES may indicate an increase in epileptic seizures caused by other etiologies. The frequency of epileptic seizures caused by etiologies other than NCC also varies across study populations. In endemic or rural regions, NCC may be one of the leading causes of epileptic seizures, whereas in non-endemic or urban regions, other etiologies such as stroke or traumatic brain injury predominate [66, 67]. In populations where non-NCC cases of epileptic seizures (e.g., cerebral tuberculosis or malaria) are common, the proportion attributable to NCC will be lower than in populations where such causes are less common. This phenomenon may be more pronounced in studies recruiting patients from clinics or hospitals where epileptic seizures of all etiologies are treated, and PWES come from both rural and urban settings.

The third source of heterogeneity relates to the context in which the study was conducted. The proportion of NCC among PWES was higher in community-based studies than in clinic/hospital-based studies. Community-based studies are generally conducted in selected communities where NCC is believed to be endemic (especially in rural communities) or where risk factors (such as poor sanitation, pig roaming, pork consumption, etc.) for *T. solium* cysticercosis prevail [68, 69]. Consequently, these types of studies tend to include groups of people who are more at risk of having NCC. If studies are conducted in regions where *T. solium* carriers are common, the results may overestimate the true burden of NCC, since *T. solium* cysticercosis tends to be clustered around parasite carriers [70]. In contrast, clinic/hospital-based studies tend to include people who are aware of the disease, able to afford brain imaging, and are willing to seek care, making them at a lower risk of NCC. Additionally, co-morbidities may be more common in patients attending clinics or hospitals. Considerable heterogeneity was also observed in the prevalence of NCC-associated epilepsy in communities. This heterogeneity may be due to the same factors described above.

Our regional estimates for AFR and AMR are slightly lower than those reported in previous meta-analyses from Sub-Saharan Africa (22%; 95% *CI:* 17%–27%) [71], and Latin America (32%; 95% *CI:* 26%–39%) [5]. In both cases, the mean estimates from previous studies fall within our 95% *BCI*s. Differences in study period likely contribute to the comparatively higher estimates reported by Bruno et al. [5]. Our review included more recent studies, whereas Bruno et al. included studies published up to 2012. However, our estimated proportion of NCC among PWES for AMR for period A is similar to that reported by Bruno et al. [5]. The higher estimate reported by Owolabi et al. (2020) may be attributable to their study inclusion criteria [71]. Specifically, their meta-analysis included studies in which NCC diagnosis was based on either serological testing, brain imaging or both, which may have inflated the estimated proportion of NCC among PWES compared with our analysis, which was restricted to studies applying brain imaging-based formal criteria for NCC diagnosis.

We did not observe any difference in NCC proportions between children and adults. This finding may contradict the general assumption that NCC is responsible for adult- or late-onset epilepsy [72–74]. This may be partially explained by our efforts to combine the data for people with different types of epilepsy and epileptic seizures into one category, PWES. In both community- and clinic/hospital-based studies, selection of the study population differed in terms of age group. We identified a number of studies that only included children, adults, or individuals of specific age groups. While the estimates from these studies may not represent the proportion of NCC among PWES in the general population, they provide important information on the NCC proportion within the selected age groups. Using data for children and adults from the same study population would improve age group-specific estimates; however, such data were rarely reported in the included articles.

Despite using a robust systematic approach, our meta-analyses were susceptible to study-level selection bias and misclassification bias. The included studies were either health center (such as clinics or hospitals) or NCC-endemic community-based, which might have biased the estimate of the NCC proportion among PWES. The lack of non-invasive diagnostic tests for NCC that could be used at the community level makes it impossible to estimate the prevalence of NCC-associated epilepsy in communities. Hence, the next best alternative was to analyse evidence from studies using formal definitions for NCC and epilepsy or epileptic seizures. Reporting estimates for community-based studies and clinic/hospital-based studies in the subgroup analyses allowed for an evaluation of the possible impact of selection bias. All studies included in this meta-analysis are cross-sectional in nature limiting the study of temporality between NCC and epileptic seizures. For the same reason, it was impossible to determine if epileptic seizures were indeed caused by NCC. A large portion of articles did not give sufficient detail on the study period, and the criteria used to diagnose NCC and epileptic seizures. Most of our included studies also lacked age- and sex-stratified data on NCC frequency, and those where data were available presented the results in strata that were not consistent across studies.

Although we followed a rigorous protocol, our study is not without limitations. First, although cysticercosis is suspected to be endemic in parts of China [75], Colombia [76], Burundi [71], and several other countries, no published literature from these countries met our inclusion criteria, which may limit the geographic representativeness of the findings. Second, almost all estimates reported in this study were based on published literature, as only one gray literature publication was eligible for data extraction, which may introduce publication bias. Third, variation in terminology and definitions used by primary studies required harmonization of diverse descriptions of study populations into the categories of PWE and PWES. While necessary for synthesis, this approach may have introduced misclassification. For example, if NCC tends to be more common in a certain type of epilepsy (e.g., active epilepsy), the proportion of NCC among PWE would be diluted.

Nevertheless, the estimates reported in this robust systematic review and meta-analysis can be used to update the global and regional burden of NCC. Updated burden estimates will help policymakers to set priorities in endemic countries. Future meta-analyses may focus on the effect of co-morbidities on the proportion of NCC among PWES.

**Disclaimer**: Raw data from this review also contributed to WHO’s burden of foodborne disease estimates. Estimates of the proportion of NCC among people with epileptic seizures may differ.

## Conclusion

In conclusion, our study provides an estimate of the proportion of NCC among PWES for different time periods, study settings, and regions. However, the findings show substantial between-study heterogeneity, reflecting important context-dependent realities. Therefore, the pooled estimates should be interpreted with care and not as universally generalizable across all endemic settings. We also highlight the decreasing trend in NCC proportion observed in different regions over time. Further research is needed to determine whether this decline in NCC proportion is real or reflects an apparent reduction resulting from changes in case definitions, study populations, advances in neuroimaging technologies for lesion detection and classification, or shifts in the underlying causes of epilepsy.

To improve the estimate of NCC among PWES, researchers are encouraged to adhere to formal standards for NCC and epileptic seizure diagnosis [13, 24]. Future studies should report age-stratified data, preferably using the age categories adopted in the Global Burden of Disease studies [77]. A prospective cohort study, including people without epileptic seizures is needed to determine the true proportion of epileptic seizures caused by NCC.

## Supplementary Information

Supplementary material 1.

## Data Availability

All data supporting the findings of this study are available within the paper and its Supplementary material 1.

## Abbreviations

AFR: African Region
AMR: Region of the Americas
*BCI*: Bayesian credible interval
*CI*: Confidence interval
CT: Computed tomography
EMR: Eastern-Mediterranean Region
EUR: European Region
FERG: Foodborne Disease Burden Epidemiology Reference Group
HICs: High-income countries
ICD: International Classification of Diseases
ILAE: International League Against Epilepsy
JBI: Joanna Briggs Institute
LMICs: Low-and-middle income countries
MRI: Magnetic resonance imaging
*n*: Number of records/studies
NCC: Neurocysticercosis
*PR*: Prevalence proportion ratio
PRISMA: Preferred Reporting Items for Systematic Reviews and Meta-Analyses
PWE: People with epilepsy
PWES: People with epileptic seizures
SEAR: South-East Asian Region
WHO: World Health Organization
WPR: Western Pacific Region
