# Supplementary material for: An updated estimate of the proportion of neurocysticercosis among people with epileptic seizures: a systematic review and Bayesian meta-analysis
Source: Infect Dis Poverty. 2026 Sep 10;15:100. doi: 10.1186/s40249-026-01501-1 (PMC13560180; doi:10.1186/s40249-026-01501-1)
Supplement: Supplementary file 1 [file 40249_2026_1501_MOESM1_ESM.docx]

**Supplementary materials**

**for**

**An updated estimate of the proportion of neurocysticercosis among people with epileptic seizures: A systematic review and Bayesian meta–analysis**

Table of Contents

[Table S1. Preferred reporting items for systematic reviews and meta–analyses (PRISMA) 2020 checklist	3](#_Toc236083198)

[Table S2. List of databases (with link) used to search articles reporting the frequency of neurocysticercosis among people with epileptic seizures	6](#_Toc236083199)

[Table S3. Description of variables used to collect data from retained literature, using Microsoft Access data extraction tool	10](#_Toc236083200)

[Table S4. Descriptive summary of studies included for final analysis	16](#_Toc236083201)

[Table S5. Summary of study–specific posterior distributions of the frequency of NCC among people with epileptic seizures	33](#_Toc236083202)

[Table S6. Summary of meta-analysis results stratified by age group (children and adults) and sex (male and female).	38](#_Toc236083203)

[Table S7. Prevalence of NCC–associated epilepsy among people living in communities	39](#_Toc236083204)

[Table S8. Sensitivity analysis results	40](#_Toc236083205)

[Fig S1. Study selection workflow for meta–analysis, subgroup meta–analysis, and meta–regression	41](#_Toc236083206)

[Fig S2. The geographical distribution of the studies on the frequency of NCC among PWES included in the current systematic review	42](#_Toc236083207)

[Fig S3. Results of subgroup meta–analyses of the proportion of NCC among people with epileptic seizures (PWES)	43](#_Toc236083208)

[Appendix S1. The Joanna Briggs Institute (JBI) critical appraisal checklist for studies reporting prevalence data	44](#_Toc236083209)

[Appendix S2. Bayesian meta–regression to estimate the level of reduction of NCC among PWES between the two periods	47](#_Toc236083210)

[References	49](#_Toc236083211)

# Table S1. Preferred reporting items for systematic reviews and meta–analyses (PRISMA) 2020 checklist

| **Section and Topic** | **Item #** | **Checklist item** | **Location where item is reported** |
| --- | --- | --- | --- |
| **TITLE** | | |  |
| Title | 1 | Identify the report as a systematic review. | Title page |
| **ABSTRACT** | | |  |
| Abstract | 2 | See the PRISMA 2020 for Abstracts checklist. | Abstracts checklist was followed to write the abstract |
| **INTRODUCTION** | | |  |
| Rationale | 3 | Describe the rationale for the review in the context of existing knowledge. | Described in “Background” section of main manuscript |
| Objectives | 4 | Provide an explicit statement of the objective(s) or question(s) the review addresses. | Described in abstract and the last part “Background” section of main manuscript |
| **METHODS** | | |  |
| Eligibility criteria | 5 | Specify the inclusion and exclusion criteria for the review and how studies were grouped for the syntheses. | Described in section “Eligibility criteria” of main manuscript |
| Information sources | 6 | Specify all databases, registers, websites, organisations, reference lists and other sources searched or consulted to identify studies. Specify the date when each source was last searched or consulted. | Name of the databases are given in “Information sources” section in main manuscript. Links of the databases are provided in “Supplementary materials”, Table S2 |
| Search strategy | 7 | Present the full search strategies for all databases, registers and websites, including any filters and limits used. | We used a similar search algorithm described under “Search strategy” section of main manuscript and Supplementary Table S2 |
| Selection process | 8 | Specify the methods used to decide whether a study met the inclusion criteria of the review, including how many reviewers screened each record and each report retrieved, whether they worked independently, and if applicable, details of automation tools used in the process. | Section “Study selection process” and “Eligibility criteria” of main manuscript |
| Data collection process | 9 | Specify the methods used to collect data from reports, including how many reviewers collected data from each report, whether they worked independently, any processes for obtaining or confirming data from study investigators, and if applicable, details of automation tools used in the process. | “Quality assessment and data collection process” section of main manuscript |
| Data items | 10a | List and define all outcomes for which data were sought. Specify whether all results that were compatible with each outcome domain in each study were sought (e.g. for all measures, time points, analyses), and if not, the methods used to decide which results to collect. | “Data item” section. All variables are listed in Table S3 in “Supplementary materials” document |
|  | 10b | List and define all other variables for which data were sought (e.g. participant and intervention characteristics, funding sources). Describe any assumptions made about any missing or unclear information. | In “Quality assessment and data collection process” and “Pooled proportion of NCC among people with epileptic seizures” section. |
| Study risk of bias assessment | 11 | Specify the methods used to assess risk of bias in the included studies, including details of the tool(s) used, how many reviewers assessed each study and whether they worked independently, and if applicable, details of automation tools used in the process. | In “Quality assessment and data collection process” section of main manuscript. Also, in Appendix S1 in “Supplementary materials” document |
| Effect measures | 12 | Specify for each outcome the effect measure(s) (e.g. risk ratio, mean difference) used in the synthesis or presentation of results. | Prevalence ratio, methods of calculation described in section “Subgroup meta–analyses and meta–regressions” |
| Synthesis methods | 13a | Describe the processes used to decide which studies were eligible for each synthesis (e.g. tabulating the study intervention characteristics and comparing against the planned groups for each synthesis (item #5)). | Described under section “Summary measures and synthesis of the results”. Article selection for meta–analysis is provided in “Supplementary materials” document, Fig S1 |
|  | 13b | Describe any methods required to prepare the data for presentation or synthesis, such as handling of missing summary statistics, or data conversions. | Described under section “Summary measures and synthesis of the results” |
|  | 13c | Describe any methods used to tabulate or visually display results of individual studies and syntheses. | Described under section “Summary measures and synthesis of the results” |
|  | 13d | Describe any methods used to synthesize results and provide a rationale for the choice(s). If meta–analysis was performed, describe the model(s), method(s) to identify the presence and extent of statistical heterogeneity, and software package(s) used. | Described under section “Summary measures and synthesis of the results” |
|  | 13e | Describe any methods used to explore possible causes of heterogeneity among study results (e.g. subgroup analysis, meta–regression). | Described under section in section “Subgroup meta–analyses and meta–regressions” |
|  | 13f | Describe any sensitivity analyses conducted to assess robustness of the synthesized results. | Described under section in section “Prior selection and sensitivity analysis” |
| Reporting bias assessment | 14 | Describe any methods used to assess risk of bias due to missing results in a synthesis (arising from reporting biases). | Not applicable |
| Certainty assessment | 15 | Describe any methods used to assess certainty (or confidence) in the body of evidence for an outcome. | JBI tool; for prevalence studies, Appendix S1 in “Supplementary materials” document |
| **RESULTS** | | |  |
| Study selection | 16a | Describe the results of the search and selection process, from the number of records identified in the search to the number of studies included in the review, ideally using a flow diagram. | Described under section, “Study selection” , and flow chart (Fig 1). |
|  | 16b | Cite studies that might appear to meet the inclusion criteria, but which were excluded, and explain why they were excluded. | Among 77 included studies six were not included in quantitative analysis (meta–analyses). They are described in section “Results of individual studies” |
| Study characteristics | 17 | Cite each included study and present its characteristics. | Supplementary materials document, Table S4 |
| Risk of bias in studies | 18 | Present assessments of risk of bias for each included study. | Supplementary Table S9 under Appendix S1 |
| Results of individual studies | 19 | For all outcomes, present, for each study: (a) summary statistics for each group (where appropriate) and (b) an effect estimate and its precision (e.g. confidence/credible interval), ideally using structured tables or plots. | “Results of individual studies” section. Also in Supplementary materials document, Table S5 |
| Results of syntheses | 20a | For each synthesis, briefly summarise the characteristics and risk of bias among contributing studies. | Provided as footnote of Table S5 in Supplementary materials document |
|  | 20b | Present results of all statistical syntheses conducted. If meta–analysis was done, present for each the summary estimate and its precision (e.g. confidence/credible interval) and measures of statistical heterogeneity. If comparing groups, describe the direction of the effect. | Meta–analysis results presented in section “pooled proportion of NCC among people with epileptic seizures”. Forest plot given in Fig 2. More statistical analysis results are provided in Supplementary Table S6 and Fig S3 |
|  | 20c | Present results of all investigations of possible causes of heterogeneity among study results. | In section “Sub–group analysis” and “Bayesian meta–regression”. Also, in “Supplementary materials” document, Appendix S2 |
|  | 20d | Present results of all sensitivity analyses conducted to assess the robustness of the synthesized results. | In “Supplementary materials” document, Table S8 |
| Reporting biases | 21 | Present assessments of risk of bias due to missing results (arising from reporting biases) for each synthesis assessed. | Not applicable |
| Certainty of evidence | 22 | Present assessments of certainty (or confidence) in the body of evidence for each outcome assessed. | Not presented as we included studies with certain evidence only |
| **DISCUSSION** | | |  |
| Discussion | 23a | Provide a general interpretation of the results in the context of other evidence. | “Discussion” section |
|  | 23b | Discuss any limitations of the evidence included in the review. | Section “Limitations of the evidence included in the review” |
|  | 23c | Discuss any limitations of the review processes used. | Section “Limitations of the review processes” |
|  | 23d | Discuss implications of the results for practice, policy, and future research. | Section “Implication of results” |
| **OTHER INFORMATION** | | |  |
| Registration and protocol | 24a | Provide registration information for the review, including register name and registration number, or state that the review was not registered. | Section “Protocol and registration” under “Methods” |
|  | 24b | Indicate where the review protocol can be accessed, or state that a protocol was not prepared. | Section “Protocol and registration” under “Methods” |
|  | 24c | Describe and explain any amendments to information provided at registration or in the protocol. | Section “Protocol and registration” under “Methods” |
| Support | 25 | Describe sources of financial or non–financial support for the review, and the role of the funders or sponsors in the review. | Section “Protocol and registration”, “Implication of results”, “Acknowledgement”, and “Funding” |
| Competing interests | 26 | Declare any competing interests of review authors. | None |
| Availability of data, code and other materials | 27 | Report which of the following are publicly available and where they can be found: template data collection forms; data extracted from included studies; data used for all analyses; analytic code; any other materials used in the review. | Codes will be open access after acceptance of the paper or upon request (Email: shah.jalal.baty@gmail.com) |

# Table S2. List of databases (with link) used to search articles reporting the frequency of neurocysticercosis among people with epileptic seizures

| Database or search platform | Link | Search string |  | Hits |
| --- | --- | --- | --- | --- |
| Africabib | <https://www.africabib.org/databases.html> | Taenia solium, Taeniasis, Taeniosis, Cysticercosis, Neurocysticercosis; Year range: 1990-2024; Level: Whole record |  | 13 |
| CAB Direct | <https://www.cabdirect.org/cabdirect/search> | (Taenia solium) OR (Taeniasis) OR (Taeniosis) OR (Cysticercosis) OR (Neurocysticercosis) AND yr:[1990 TO 2024] |  | 6,701 |
| Embase | [https://www.embase.com/landing?status=gray](https://www.embase.com/landing?status=grey) | (Taenia solium or Taeniasis or Taeniosis or Cysticercosis or Neurocysticercosis).af; limit to yr="1990 -Current"; limit to "remove medline records" |  | 2,539 |
| Global Health | <https://www.cabdirect.org/globalhealth> | (Taenia solium or Taeniasis or Taeniosis or Cysticercosis or Neurocysticercosis).af; limit to yr="1990 -Current" |  | 6,131 |
| Google Scholar^a^ | <https://scholar.google.ca/> | Taenia solium OR Taeniasis OR Taeniosis OR Cysticercosis OR Neurocysticercosis; Custome range: 1990-2024 |  | 25,000 |
| Hispanic American Periodicals Index | <https://hapi.ucla.edu/> | Taenia solium or Taeniasis or Taeniosis or Cysticercosis or Neurocysticercosis; Publication year: 1990-2024 |  | 0 |
| INASP journal online project^b^ | [https://www.inasp.info/project/journals–online–project](https://www.inasp.info/project/journals-online-project) | Not applicable |  | 1,035 |
| *–African Journal Online* | <https://www.ajol.info/index.php/ajol> | AJOL=(Taenia solium, Taeniasis, Taeniosis, Cysticercosis, Neurocysticercosis) |  | 965 |
| *–Vietnam Journals Online* | https://vjol.info.vn/ | (Taenia solium, Taeniasis, Taeniosis, Cysticercosis, Neurocysticercosis) and (Taenia solium OR Taeniasis OR Taeniosis OR Cysticercosis OR Neurocysticercosis)  (Taenia solium HOẶC Bệnh sán dây HOẶC Bệnh sán dây HOẶC Bệnh sán dây thần kinh) and (Taenia solium, Bệnh sán dây, Bệnh sán dây, Bệnh sán dây thần kinh); Year: 2020 January 01-2023 December 31 |  | 0 |
| *–Bangladesh Journals Online* | https://www.banglajol.info/ | Taenia solium OR Taeniasis OR Taeniosis OR Cysticercosis OR Neurocysticercosis; Year: 1994 January 01-2023 December 31 |  | 12 |
| *–Central America Journals Online* | <https://www.camjol.info/> | Taenia solium OR Taeniasis OR Taeniosis OR Cysticercosis OR Neurocysticercosis; Year: 1990 January 01-2023 December 31 |  | 6 |
| *–Mongolia Journals Online* | https://www.mongoliajol.info/ | Taenia solium OR Taeniasis OR Taeniosis OR Cysticercosis OR Neurocysticercosis; Year: 1994 January 01-2023 December 31 |  | 1 |
| *–Nepal Journals Online* | https://www.nepjol.info/ | Taenia solium OR Taeniasis OR Taeniosis OR Cysticercosis OR Neurocysticercosis; Year: 1990 January 01-2023 December 31 |  | 51 |
| Latin American Open Archives Portal (LAOAP) | <http://lanic.utexas.edu/project/laoap/> | Taenia solium, Taeniasis, Taeniosis, Cysticercosis, Neurocysticercosis |  | 0 |
| MEDLINE^c^ | <https://www.nlm.nih.gov/medline/medline_overview.html> | Taenia solium/ Taeniasis/Taeniosis.mp. or Cysticercosis/Neurocysticercosis; limit to yr="1990 - Current" |  | 5,456 |
| PubMed | <https://pubmed.ncbi.nlm.nih.gov/> | (((((Neurocycticercosis/epidemiology[MeSH Terms]) OR (Taenia solium)) OR (Taeniasis)) OR (Taeniosis)) OR (Cysticercosis)) OR (Neurocysticercosis); Year: 1990-2023 |  | 7,057 |
| SciELO–Scientific Electronic Library Online | <https://scielo.org/en> | (*taenia solium) OR (taeniasis) OR (taeniosis) OR (cysticercosis) OR (neurocysticercosis) AND year_cluster:("2004" OR "2012" OR "2017" OR "2002" OR "2006" OR "2007" OR "2014" OR "2018" OR "2016" OR "2000" OR "2013" OR "2015" OR "2003" OR "2010" OR "2022" OR "2020" OR "2001" OR "1990" OR "2005" OR "2009" OR "2011" OR "2019" OR "2021" OR "1996" OR "1999" OR "1994" OR "1995" OR "2008" OR "1991" OR "1992" OR "1997" OR "1998" OR "1993" OR "2023")  *(taenia solium) OR (teniasis) OR (taeniosis) OR (cisticercosis) OR (neurocisticercosis)  *(taenia solium) OR (teníase) OR (teniose) OR (cisticercose) OR (neurocisticercose) |  | 495 |
| Scopus | [www.scopus.com](http://www.scopus.com/) | TITLE-ABS-KEY (Taenia solium OR Taeniasis OR Taeniosis OR Cysticercosis OR Neurocysticercosis); Years: 1990-2024 |  | 9,500 |
| Web of Science | <https://www.webofscience.com/> | ((((ALL=(Taenia solium)) OR ALL=(Taeniasis)) OR ALL=(Taeniosis)) OR ALL=(Cysticercosis)) OR ALL=(Neurocysticercosis); Publication date: 1990-01-01 to 2024-01-01 |  | 6,515 |
| Oaister | <https://oaister.on.worldcat.org/discovery> | kw:(Taenia solium) OR kw:(Taeniasis) OR kw:(Taeniosis) OR kw:(Cysticercosis) OR kw:(Neurocysticercosis) AND (yr:1990..2024) |  | 1,580 |
| GrayGuide | http://greyguiderep.isti.cnr.it/userarea.php?langver=en | Taenia solium, Taeniasis, Taeniosis, Cysticercosis, Neurocysticercosis; Year: 1990-2024 |  | 0 |
| WHO–African Index Medicus (AIM) | <https://www.globalindexmedicus.net/biblioteca/aim/> | tw:((tw:(taenia solium)) OR (tw:(taeniasis)) OR (tw:(taeniosis)) OR (tw:(cysticercosis)) OR (tw:(neurocysticercosis))) AND ( collection_gim:("AIM")) AND (year_cluster:[1990 TO 2024]) |  | 9 |
| WHO–Index Medicus for Eastern Mediterranean Region (IMEMR) | <https://www.globalindexmedicus.net/biblioteca/imemr/> | mtw:((tw:(taenia solium)) OR (tw:(taeniasis)) OR (tw:(taeniosis)) OR (tw:(cysticercosis)) OR (tw:(neurocysticercosis))) AND ( collection_gim:("IMEMR")) AND (year_cluster:[1990 TO 2024]) |  | 59 |
| WHO–Latin America & the Caribbean Literature on Health Sciences (LILCAS) | <https://www.globalindexmedicus.net/biblioteca/lilacs/> | tw:((tw:(taenia solium)) OR (tw:(taeniasis)) OR (tw:(taeniosis)) OR (tw:(cysticercosis)) OR (tw:(neurocysticercosis))) AND ( collection_gim:("LILACS")) AND (year_cluster:[1990 TO 2024]) |  | 887 |
| WHO Library (WHOLIS) | https://kohahq.searo.who.int/ | Taenia solium or Taeniasis or Taeniosis or Cysticercosis or Neurocysticercosis; Publication date range: 1990-2024 |  | 2 |
| WHO–Index Medicus for South–East Asia Region (IMSEAR) | <https://www.globalindexmedicus.net/biblioteca/imsear/> | tw:((tw:(taenia solium)) OR (tw:(taeniasis)) OR (tw:(taeniosis)) OR (tw:(cysticercosis)) OR (tw:(neurocysticercosis))) AND ( collection_gim:("IMSEAR")) AND (year_cluster:[1990 TO 2024]) |  | 355 |
| WHO–The Western Pacific Region Index Medicus (WPRIM) | <https://www.globalindexmedicus.net/biblioteca/wprim/> | tw:((tw:(taenia solium)) OR (tw:(taeniasis)) OR (tw:(taeniosis)) OR (tw:(cysticercosis)) OR (tw:(neurocysticercosis))) AND ( collection_gim:("WPRIM")) AND (year_cluster:[1990 TO 2024]) |  | 297 |
| Total initial hits |  |  |  | **74,666** |
| Accessible titles of the records^a^ |  |  |  | **50,663** |
| Initial removal of duplicate records |  |  |  | **2,920** |
| Records uploaded to Covidence |  |  |  | **47,743** |

^a^There were 25,000 initial hits in Google Scholar, but 997 citations were accessible for screening.

^b^This project also include – Philippines Journals Online (the website was not found on the day of article search), and Sri Lanka Journals Online (article searching interface was not found on the website).

^c^Although MEDLINE is now the part of PubMed, we did literature search on MEDLINE using Ovid platform from library–website of the Université de Montréal, Canada.

# Table S3. Description of variables used to collect data from retained literature, using Microsoft Access data extraction tool

| Field name | Variable | Data Type | Description |
| --- | --- | --- | --- |
| Form: *Reviewer* |  |  |  |
| Identification reviewer/article | | | |
| E_DateAb | Date of Abstraction | Date/Time | Short Date = MM/DD/YYYY, date of the data extraction |
| OUHSCID | Article ID | Number | Unique 6 digits ID for each included study. ID starts from 300001 |
| ReviewerID | Reviewer id | Number | Auto populated field |
| First_name | First name of reviewer | Short Text | First name of the person who reviews the article |
| Form: *Epidemiology* | |  |  |
| Identification |  |  |  |
| OUHSCID | NA | Number | Auto populated field |
| ReviewerID | NA | Number | Auto populated field |
| Study type/time periods | | | |
| E_StuDesign | Study design | Short Text | Study designs: "Case Report ( < 20)"; "Case Series ( >= 20)"; "Cross–sectional"; "Ecological"; "Case–Control"; "Cohort"; "Randomized Clinical Trial"; "Randomized Community Trial"; "Review"; "Editorial". Selection of study design was based on the nature of NCC–frequency data collection, NOT based on what was described by the author. |
| E_YrStudy | Study year | Number | Long Integer. Year that the study began |
| E_StudyDur | Study duration | Number | Long Integer. Duration of the study. Input was either number of days or months or years. |
| E_DurUnit | Duration unit | Short Text | Text Picklist. Unit for duration of study: "Years"; "Months"; "Days"; "Unk". Unk was selected if data was not found. |
| Frequency |  |  |  |
| E_Frequency | Frequency | Short Text | Text Picklist. Type of frequency reported: "Point prevalence"; "Period prevalence"; "Incidence Rate", "Risk”, “Proportion_study” |
| E_FreqUnit | Frequency unit | Short Text | Free text. Unit of frequency reported |
| Population/Generalizability | | | |
| E_Sampling | Sampling | Short Text | Picklist: "Census";"SRS"; "Cluster_Strata"; "Non_Probalistic";"Unk". In case of “Unk” = Unknown, study was excluded; Other(specify) |
| E_TgtPopulation | Target population | Short Text | Description_Target Population.Pickup list:1°Comunity; 2°Clinic_OUTpatientSE; 3°Hosp_OUTpatientSE; 4°Hosp_INpatientSE 5°Hosp_INpatientCNS; 6° Hosp_INOUTpatient; 7° Autopsies; 8°"Comunity_Seropos";9°Comunity_Seroneg; 10°Comunity_Manifest;11°Other_specify; |
| E_TargetPop | Target population | Short Text | Free text. What was the Target Population for this Study? The target population from which the study population was drawn. |
| E_TgtPopSz | Target population size | Number | Number in Target Population: the total number in the Target Population. If not stated in paper, kept blank. |
| E_StudySize | Study size | Number | Number of Study Participants: the total number of Study Participants. Studies having N < 20 were Excluded. |
| E_EligCrit | Eligibility criteria | Short Text | Were eligibility criteria the same for groups being compared? Picklist: "Yes"; "No"; "Unk"; "Unk" = Not enough information given to know. If No, then the study was Excluded. |
| E_SelSame | Selected similarly | Short Text | Were groups compared selected similarly from the same Target Population & were response proportions similar in the 2 groups? Picklist: "Yes";"No";"Unk". "Unk" = Not enough information given to know. If No, then the study was Excluded. |
| E_RepTgt | Representative | Short Text | Are groups representative of the same Target Population? Allows to evaluate Selection Bias. Picklist: "Yes"; "No"; "Unk". "Unk" = Not enough information given to know. If No, then the study was Excluded. |
| E_RepGen | Represent source pop | Short Text | Are diseased and non–diseased representative of the Source Pop, the General population of the Area in which study occurs. Picklist: "Yes"; "No"; "UnK". Unk = Not enough information given to know. If yes, study results are generalizable to the Source Pop |
| Selection bias |  |  |  |
| E_SourcePop | Source population | Short Text | Free text. What was the Source Population for this Study?. Plan to generalize to the district or at most a region. Our thought = the study area may not represent a large area, a state, or a country. |
| E_StudyPop | Study population | Short Text | Free text: Description of the study population |
| Diagnosis |  |  |  |
| E_Investigations | Investigation | Short Text | What type of investigations were done (Biology, Imaging). Pick up list: "NA"; “Imaging_only”;”Serology_only”;”ImagingAndSerology”;"Biopsy"; "Autopsy";”Other_specify” |
| Biology |  |  |  |
| E_DxInfection | Non–imaging diag. test | Short Text | Serological, Histological or coprological test used. (Ag, Ab, purgation, stools examination). PickList "Yes"; "No"; "Unk" = Not enough information given to know |
| E_DxTest | Name of test used | Short Text | Test used. Picklist. "AbELISA"; "Westernblot (EITB)"; "AgELISA"; "Ag&Ab ELISA"; "AgELISA&EITB"; "AbELISA&EITB"; "copro"; "copro–Ag"; "copro&copro–Ag"; "copro&purgation"; purgation"; "copro–antigen & PCR"; "histology"; "autopsy"; "other (specify)". |
| Other_Dx1 | Other diag test | Short Text | Free text. any other diagnosis test. Default: "NA" |
| Whose_DX |  | Short Text | Whose diagnosis? Multiple entries allowed. Check list: "NA"; "All Sampled"; "SubSamplePop";"Epilepsy";"Seizure";"Headache"; "Other Neurological Disorder" |
| E_LabTest | Lab Test | Short Text | Check list /Ag &Ab are ELISA/Many entries possible: Ab_MetCrudeExtracts_Tspp,Ab_MetPurifiedFr_Tsolium,Ab_MetRecombinant_Tsolium,Ab_Oncospheres,Ab_Both,Ab_Unknown,Ab_Other,Ag_MonclonalBrandt_Tsolium,Ag_Unknown,Ag_Other,Westernblot (EITB),Other_Labtest,NA |
| Specify_LabTest/ E_Other_LabTest | Specify other Lab Test | Short Text | Free text. any other diagnosis test. Default: "NA" |
| Imaging |  |  |  |
| Imaging | Imaging | Short Text | Was imaging used for the diagnosis (does not apply to taeniasis and cysticercosis). PickList: "Yes"; "No"; "Unkn"; "NA". NA is used for taeniasis and cysticercosis. "Unk" = Not enough information given to know. |
| Whose_images | Study population | Short Text | Who was sent for Imaging? Multiple entries allowed. Check list: "NA"; "All Sampled"; "Seropositive";"Epilepsy";"Seizure";"Headache";"Seronegative"; "Other Neurological Disorder" |
| TypeImaging | Type of imaging | Short Text | Type of imaging test used. Multiple Picklist: "NA"; "MRI"; "CT–scan"; "X–ray"; "EEG"; "ultrasound"; "Other (specify)" |
| E_CNSDisorder | Neuro–disorder | Short Text | Free Text: Specify the CNS disorder |
| OtherImage | Other imaging | Short Text | Free text. any other Imaging technique. Default: "NA" |
| Seroneg_imaging | Imaging of seronegative people | Short Text | Text. Picklist: "Yes"; "No"; "Unk"; Imaging of seronegative participants |
| Seroneg_imaging_Sample | Sampling of seronegative people | Short Text | Text. Picklist:"Census"; "Probability"; "Non_Probability"; "Unk". "NA"; Sampling of the seronegative people who had an imaging test |
| Form: *Definition/Inclusion/Notes* | | |  |
| Definition |  |  |  |
| E_Investigation | NA | Short Text | Auto populated |
| NCC_Diag_Criteria | Diagnostic criteria used: NCC | Short Text | Pick list. Diagnostic criteria or reference used for NCC–diagnosis. Multiple selection possible. |
| If_Ndiag(Ref)_Put_DOI | Link of diagnostic criteria | Short Text | If NCC diagnostic criteria was based on any reference input the DOI of that reference. |
| Definition_NCC | Description of diagnostic criteria | Long Text | Free text. Description of the definition of NCC cases. |
| Epilepsy_Diag_Criteria | Diagnostic criteria used: epilepsy | Short Text | Pick list. Diagnostic criteria or reference used for epilepsy or seizure. Multiple selection possible. |
| If_Ediag(Ref)_Put_DOI | Link of diagnostic criteria | Short Text | If epilepsy/seizure diagnostic criteria was based on any reference, input the DOI of that reference. |
| Def_Epilepsy | Definition of epilepsy | Long Text | Free text. Description of the definition of epilepsy or seizure. |
| Headache_Diag_Criteria | Diagnostic criteria used: headache | Short Text | Pick list. Diagnostic criteria or reference used for headache. “Not_Provided”, “Neuroexpert_Opinion”, “Reference” |
| If_Hdiag(Ref)_Put_DOI | Link of diagnostic criteria | Short Text | If headache diagnostic criteria was based on any reference input was the DOI of that reference. |
| Def_headache | Definition Headache | Long Text | Definition of Headache |
| Form: *JBI checklist* |  |  |  |
| Sample_Frame | JBI: Question 1 | Short Text | 1. Was the sample frame appropriate to address the target population? |
| Sampling | JBI: Question 2 | Short Text | 2.Were study participants sampled in an appropriate way? |
| Sample_Size | JBI: Question 3 | Short Text | 3.Was the sample size adequate? |
| Subject_Setting_Description | JBI: Question 4 | Short Text | 4.Were the study subjects and the setting described in detail? |
| Data_Analysis_Coverage | JBI: Question 5 | Short Text | 5.Was the data analysis conducted with sufficient coverage of the identified sample? |
| Outcome_Measure | JBI: Question 6 | Short Text | 6.Were valid methods used for the identification of the condition? |
| Outcome_Measure_ForALL | JBI: Question 7 | Short Text | 7.Was the condition measured in a standard, reliable way for all participants? |
| Stat_Analysis | JBI: Question 8 | Short Text | 8.Was there appropriate statistical analysis? |
| Response_Rate | JBI: Question 9 | Short Text | 9.Was the response rate adequate, and if not, was the low response rate managed appropriately? |
| Overall_Appraisal | Quality of data | Short Text | Decision: "Include";"Exclude";"Seek further info" |
| Inclusion |  |  |  |
| Include | Final decision | Short Text | Decision of Inclusion or non inclusion of the article. Pick up list: " Yes"; "No"; "Undecided" |
| Reason_out | Reason of exclusion | Short Text | Reason_Non inclusion of the article.Tick correct answers.Multiple entries: "Non_agent";"Animal";"No_Frequency";"Study_Period";"Case_report";"Review";"Editorial";"Case_Series";"Eligibility_Criteria";"No_Imaging";"Selection_bias";"No_NCCDefinition" |
| Notes |  |  |  |
| Notes | Notes on article | Long Text | Input additional information you think is important, pertinent, or worthy of discussion regarding the paper. |
| Quantitative |  |  |  |
| OUHSCID | NA | Number | Autogenerated |
| First_name | NA | Short Text | Autogenerated |
| Sero_status | Sero–status of study population | Short Text | Sero status of the patient group |
| Manifestation | Manifestations among study population | Short Text | Pick up list: Epilepsy; Lifelong epilep; Active epilep; Incident epilep; Single seizures; Recurrent seizures; Hydrocephalus; ICP = Increased intracranial pressure; Headache; ICP_Headache = Increased intracranial pressure and Headache; Dementia; None; Other |
| Agegp | Age group | Short Text | Pick up list: All; Adult; Elderly; Children |
| Min_Age | Minimum age | Number | Minimum age of the age group chosen |
| Max_Age | Maximum age | Number | Maximum age of the age group chosen |
| Age_range | Age range | Short Text | Age range |
| Mean_age | Mean age | Number | Average age of study population |
| Gender | Gender | Short Text | Gender of the group |
| Nbr_asked_imaging | Target population | Number | Number of people asked for imaging |
| Nbr_w_imaging | Study population | Number | Number of people with imaging |
| Nbr_w_NCC | Positive cases | Number | Number of people with NCC |
| Nbr_w_Scolex | # of people with scolex | Number | Number of people with cystic lesions showing the scolex on CT or MRI |
| Nbr_histology | Diagnosis based on histology/biopsy | Number | Number of people with cystic lesions showing the scolex at histology / biopsy |
| Nbr_fundoscopy | Diagnosis based on fundoscopy | Number | Number of people with direct visualization of subretinal parasites by funduscopic examination |
| Nbr_w_colloidal_cyst | People with colloidal cyst | Number | Number of people with colloidal cysts |
| Nbr_w_calcified_cyst | Calcified NCC | Number | Number of people with calcified cysts |
| Nbr_enhancing_lesions | People with enhancing lesion | Number | Number of people with enhancing lesions |
| Nbr_minor_CT_lesions | Other imaging lesion | Number | Number of people with lesions compatible with NCC on neuroimaging studies (e.g., CT or MRI showing hydrocephalus or abnormal enhancement of the leptomeninges) |
| Nbr_CC_outside_CNS | Cysticercosis | Number | Number of people with cysticercosis outside the CNS (e.g., histologically confirmed subcutaneous or muscular cysticercosis or direct visualization of cysticerci in the anterior chamber of the eye) |
| Nbr_CSF_ELISA_performed | Serology | Number | Number of people with cerebral spinal fluid ELISA performed |
| Nbr_CSF_positive | Positive in serology | Number | Number of people with positive CSF ELISA |
| Nbr_NCCAutopsy | Diagnosis by autopsy | Number | Number of people with NCC whose corpse underwent an autopsy |
| Nbr_Postmortem | Diagnosis based on PM report | Number | Number of corpses that underwent an autopsy |
| Nbr_ChartReviewed | Diagnosis based on medical record | Number | Number of medical/clinical/hospital discharge/death report reviewed |
| Comment | Any extra information | Long Text | Any comment on this entry |
| Computable | Estimating the proportion | Yes/No | Can the prevalence be calculated or back–calculated? |

# Table S4. Descriptive summary of studies included for final analysis

| **Country, year(s) of study** | **Reference (language)** | **Design** | **Source population** | **Target population** | **Study population** | **NCC diagnosis/ Definition** | **Diagnosis/definition of manifestation** | **Types of epileptic seizures** | **Types of frequency** | **Sampling** | **Study *with* formal definition?^c^** |
| --- | --- | --- | --- | --- | --- | --- | --- | --- | --- | --- | --- |
| Bhutan, 2014–15 | [1](English) | Case series | People of all 20 districts of Bhutan | 205 PWE^1^ who had both EITB and MRI | 205 | According to [2] | Epilepsy: patient with a witnessed or self–reported history of ≥ 1 seizures with no evidence of a competing diagnosis such as cardiac dysrhythmia or exclusively febrile seizures | Epilepsy | Proportion of NCC among PWE | Census | Yes |
| Bolivia,  1994 | [3] (English) | Cross–sectional | 55,675 people of rural area of Cordillera province, Santa Cruz Department | 124 people diagnosed as having prevalent epilepsy | 105 who underwent CT– scan | According to [4] | Epilepsy was diagnosed based on [5] | Epilepsy | Prevalence of NCC among PWE | Census/door–to–door survey | Yes |
| Brazil,  NA–NA | [6] (English) | Case series | Catchment population who had access to the neurology clinic | 580 chronic epileptics who attended as outpatients of a neurology clinic | 210 chronic epileptics who were followed for 3 years | (1) CT–scan findings compatible to NCC [7, 8]; (2) positive CSF immunological test for NCC; and/or (3) surgical diagnosis of NCC | Chronic epilepsy: ≥ 2 seizures in the six months before evaluation; Seizure classified according to [5] | Chronic epilepsy | Proportion of NCC among patients with chronic epilepsy | Random sampling | Yes |
| Brazil,  NA–NA | [9] (English) | Case series | People attending to the Hospital de Clínicas de Porto Alegre | 731 PWE who participated in a cohort study | 659 PWE who had CT–scan | According to [10] | Epilepsy: defined according to [11, 12] | Epilepsy | Proportion of calcified NCC among patients (age: > 15 years) with epilepsy | Census | Yes |
| Brazil,  1978–90 | [13] (Portuguese) | Case series | People of catchment area of the Computerized Tomography and  Magnetic Resonance Center (CETAC), Curitiba, Parana | 1000 consecutive patients with history of epileptic seizure who had CT | 1000 | Presence of intraparenchymal cystic or calcified lesion or racemose lesions | Seizures were classified according to [5] | Seizures | Proportion of NCC among patients with epileptic seizure | Census | Yes |
| Brazil,  1995–96 | [14] (Portuguese) | Case series | People attending the Hospital Regional de Chapecó,Santa Catarina | 100 patients hospitalized due to epileptic seizures | 58 patients with epileptic seizure who had CT–scan | CT diagnostic criteria suggested by [15] | Medical record review | Seizures | Proportion of NCC among patients with epileptic seizure** | Census | No |
| Brazil,  2005 | [16] (Portuguese) | Case series | People attending the Celso Pierro Hospital and the Maternity Hospital of the Pontifical Catholic University | 102 consecutive patients with seizure (although author mentioned as “epilepsy” based on medical chart–review) | 102 with CT–scan | Intracranial calcification suggestive to NCC diagnosed according to [17] | Medical record review | Seizures | Proportion of intracranial calcification suggestive of NCC among patients with Seizure** | Census | No |
| Brazil,  2009–11 | [18] (Portuguese) | Case series | People attending the Hospital das Clínicas of the Federal University of Minas Gerais | 502 patients with epilepsy followed up in the hospital & had MRI/CT–scan | 502* | According to [4] | Classification of seizures type based on [5] | Epilepsy | Proportion of NCC among patients with epilepsy | Census | Yes |
| Brazil,  2013 | [19] (English) | Case series | People attending the University of Campinas Hospital | 268 patients with epilepsy who attended epilepsy clinic & had MRI/ CT–scan | 268 | According to [2] | Epilepsy was diagnosed as per [20] and classified based on [21] | Epilepsy | Proportion of NCC among patients with epilepsy | Census | Yes |
| Burkina Faso 2007 | [22] (English) | Cross–sectional | 888 interviewed people of Batondo, Pabre, Nyonyogo villages of Burkina Faso. Total population was approximately 8,500 | 72 people with confirmed epilepsy | 68 accepting CT–scan | According to modified criteria of [4] | Epilepsy: ≥ 2 unprovoked seizures separated by ≥ 24 h. Seizures were classified according to [23] | AE (83%, defined as 1 epileptic seizures or use of antiepileptic drug in 3 years) | Prevalence of NCC among people with epilepsy | Cluster random sampling & self–identified | Yes |
| Cameroon, 1999–05 | [24] (French) | Case series | People attending the Yaoundé central hospital | 129 consecutive patients with epilepsy | 75 with brain scan† | Medical record review, assumed NCC was diagnosed by a neurology expert | Medical record review, assumed epilepsy was diagnosed by a neurology expert | Epilepsy | Proportion of NCC among patients with epilepsy** | Census | No |
| Colombia,  1995 | [25] (English) | Case series | People who attended 9 outpatient clinics of Instituto Neurologico de Antioquia  (INDEA), Medellin | 643 people with epilepsy | 546 PWE who had CT scan | ≥ 1 living or degenerating cysticercosis lesions, or ≥ 2 calcifications nodular or annular, smaller than 1 cm calcification | Epilepsy: ≥ 1 afebrile seizures unrelated to acute metabolic disorders or to discontinuation of drugs or alcohol | Epilepsy | Proportion of NCC among patients with epilepsy | Census | Yes |
| Côte d'Ivoire, 1994–96 | [26] (French) | Case series | People from catchment area of Saint–Anne–Marie international polyclinic | 205 people with symptomatic epilepsy who had CT–scan | 205 | Medical record review, assumed NCC was diagnosed by a neurology expert | Epilepsies are chronic condition of various etiologies characterized by repetition of seizures resulting from a discharge of cerebral neurons, whatever the clinical or paraclinical symptoms possibly associated [27] | Epilepsy | Proportion of NCC among patients with epilepsy** | Census | No |
| Côte d'Ivoire, 2015–17 | [28] (English) | Case series | People attending the General Hospital Adjamé and the Cocody and Yopougon University Hospitals | 403 patients visited the neurology units for the first time for epileptic seizures | 114 epileptic patients who had CT–scan results in their medical record | According to [10] | Epileptic patients: at least two spontaneous epileptic seizures within an interval of more than 24 h [20] | Epilepsy | Proportion of NCC among patients with epilepsy | Census | Yes |
| Ecuador,  1992 | [29] (English) | Cross–sectional | 2,723 Resident of San del Lago, Andean, | 31 individuals with active epilepsy | 26 who accepted CT–scan | According to [30, 31] | AE was diagnosed according to [32] | AE (100%, defined as 1 epileptic seizure in last 5 years) | Prevalence of NCC among people with active epilepsy | Census/ door–to–door survey | Yes |
| Ecuador, 1986–90 | [33] (English) | Case series | People attending the Hospital Luis Vernaza | 225 consecutive patients late–onset epilepsy with CT–scan | 225 | Diagnosed by neurologist | Assumed epilepsy was diagnosed by neurologist. Late–onset epilepsy: seizures starting after 25 years of age. Seizures were classified according to [5] | Late–onset epilepsy | Proportion of NCC among patients with late–onset epilepsy | Census | No |
| Ecuador,  2003 | [34] (English) | Cross–sectional | 2,548 residents in 445 Atahualpa village | 24 people with epilepsy | 19 who had CT–scan | According to [4] | Epilepsy was diagnosed according to [5, 35] | Epilepsy | Prevalence of NCC among PWE | Census/door–to–door survey | Yes |
| Ecuador, 1990–09 | [36] (English) | Case series | People attending the Hospital–Clinica Kennedy, Guayaquil, Ecuador | 446 consecutive patients with late–onset epilepsy | 431 who had MRI/CT–scan | According to [4] | Assumed epilepsy was diagnosed by neurologist. Late–onset epilepsy: recurrent seizures starting after 20 years of age | Late–onset epilepsy | Proportion of NCC among patients with late–onset epilepsy | Census | Yes |
| Ecuador, 2012–15 | [37] (English) | Cross–sectional | 1,604 surveyed adult (age: ≥ 20 years) residents of Atahualpa | 41 adult PWE | 39 adult PWE who accepted CT–scan | According to [10] & [38] | Epilepsy: recurrent non–provoked seizures. Epilepsy was diagnosed by neurologist | Epilepsy | Prevalence of NCC among adult people with epilepsy | Census/door–to–door survey | Yes |
| Ecuador, 2012–15 | [37] (English) | Cross–sectional | 1604 surveyed adult (age: ≥ 20 years) residents of Atahualpa | 7 adult people with single seizure (occurred > 5 years before survey) | 7 adult people with single–seizure who underwent CT–scan | According to [10] & [38] | Single seizure was diagnosed by neurologist | Single seizure | Prevalence of NCC among adult people with single seizure | Census/door–to–door survey | Yes |
| Honduras,  1997 | [39] (English) | Cross–sectional | 6,473 residents of Salama County | 100 people with active epilepsy | 90 who accepted CT–scan | According to [4] | The definitions and classifications for epileptic seizures proposed by [5, 35] | AE (100%, defined as any seizures in last 5 years) | Prevalence of NCC among patients with active epilepsy | Census/door–to–door survey | Yes |
| Honduras, 1997–05 | [40] (English) | Cross–sectional | 5,609 inhabitants of Salama County, Olancho who were evaluated | 36 people with active epilepsy | 33 who accepted CT–scan | Diagnosed by neurophysiologists | Epilepsy & seizures: defined & classified according to [5, 32, 35] | AE (100%, defined as ≥ 2 seizures in last 5 years) | Prevalence of NCC among people with active epilepsy‡ | Census | No |
| India,  1992–93 | [41] (English) | Case series | Children attended to the National Institute of Mental Health and Neuro Sciences (NIMHANS), Bangalore | 198 children who registered at NIMHANS for Simple partial seizures | 198 who underwent CT–scan | According to [30] | Simple partial seizure was defined according to [5] | Seizures | Proportion of NCC among children with simple partial seizures | Census | Yes |
| India,  1995–99 | [42] (English) | Case series | Children from catchment area of Jawaharlal Institute of Postgraduate  Medical Education and Research (JIPMER), Pondicherry | 150 consecutive children presented with simple or complex partial seizures and had CT–scan | 150 | According to [4] | Assumed seizures were diagnosed by neurology expert | Seizures | Proportion of NCC among children with partial seizures | Census | No |
| India,  1995–01 | [43] (English) | Case series | People from Punjab, Rajasthan and Himachal Pradesh | 1,026 consecutive patients with seizures or epilepsy presenting to a teaching hospital | 936 PWE who had CT/MRI within 7 days of a seizure | (i) ≥ 1 hypodense (upon CT) or CSF  intensity (upon MRI) cystic lesions with or without an eccentric nodule, (ii) ≥ 1 ring or disk–enhancing lesions, or (iii) ≥ 1 calcified nodules at the cortical–subcortical interface | Different types of seizures or epilepsy were defined according to [32] | Single seizure & Epilepsy | Proportion of NCC among people with seizures, and people with epilepsy | Census | Yes |
| India,  2003–04 | [44] (English) | Cross–sectional | 50,617 residents of Vellore district, Tamil Nadu | 194 people with active epilepsy | 162 people with active epilepsy who accepted | According to [4] | AE was diagnosed according to [45] | AE (100%, defined as ≥ 1 seizures within 5 years) | Prevalence of NCC among patients with active epilepsy | Census/door–to–door survey | Yes |
| India,  NA–NA | [46] (English) | Case series | Children attending the Nehru Hospital, Chandigarh | 124 consecutive children (age: ≤ 12 years) with partial seizures | 100 children with partial seizures who had CT | A ring– or disc enhancing lesion with perifocal edema on CT scan and a rising titer ( ≥ 1:512) on serology; & brain autopsy | Partial seizure was defined according to [5] | Seizures | Proportion of NCC among children with partial seizures** | Census | Yes |
| India,  NA–NA | [47] (English) | Cross–sectional | 1,028 individuals belonging to every fifth household of Basmatia tea estate community, Dibrugarh district | 53 patients with active epilepsy | 45 who accepted CT–scan | According to [2, 48] | Epilepsy: according to [49]; AE: according to [32] | AE (100%, defined as ≥ 1 seizures within 5 years) | Prevalence of NCC among people with active epilepsy | Systematic random sampling | Yes |
| India,  NA–NA | [50] (English) | Case series | Catchment population of an Indian hospital | 129 consecutive patients with new‑onset seizures | 126 who had MRI & EEG | NCC was diagnosed by radiologists | Seizures: according to [5] | Seizures | Proportion of NCC among patients with new–onset seizures | Census | No |
| India,  NA–NA | [51] (English) | Cross–sectional | 2,209 surveyed people Chattah zone of district Jammu | 25 people with active epilepsy | 25 who had MRI/CT–scan | According to [4] | AE: according to [32] | AE (100%, defined as ≥ 1 seizures within 5 years) | Prevalence of NCC among people with active epilepsy | Village cluster/ door–to–door survey | Yes |
| India,  NA–NA | [52] (English) | Cross–sectional | 14,086 people of Chakrata block of Dehradun district in Uttarakhand | 141 people with active epilepsy | 141 who had CT– scan | According to [4] | Epilepsy/AE: according to [32] | AE (100%, defined as ≥ 1 seizures within 5 years) | Prevalence of NCC among people with active epilepsy | Census/door–to–door survey | Yes |
| India,  NA–NA | [53] (English) | Cross–sectional | Residents of pig farming community in Mohanlalganj ,Lucknow district, Uttar Pradesh | 60 PWE | 60* who accepted MRI | According to [4, 54] | Seizures classification according to [32] | Epilepsy | Prevalence of NCC people with epilepsy | Census/door–to–door survey | Yes |
| India,  1995–01 | [55] (English) | Case series | People attending the Dayanand Medical College, Ludhiana | 308 consecutive patients (age: > 12 years) with new–onset seizures (both acute symptomatic or unprovoked) | 308 who had MRI/CT–scan | (1) single or multiple actively degenerating brain parenchymal cysticerci with surrounding edema, (2) single or multiple calcific lesions consistent with the inactive stage of cysticerci, or (3) any combination of (1) and (2) with no other perceived cause for the seizures | Seizures classification according to [56] | Seizures | Proportion of NCC among patients with new–onset seizures | Census | Yes |
| India,  2001 | [57] (English) | Cross–sectional | People from 68 villages of Wes Godavari district, Andhra Pradesh, India | 462 PWE | 379 PWE who had CT–scan | According to [4] | Epilepsy: ≥ 2 epileptic seizures 24 hours apart [20] | Epilepsy | Prevalence of NCC among people with epilepsy | Village cluster/ door–to–door survey | Yes |
| India,  2001–02 | [58] (English) | Case series | Children (age: ≤ 12 years) of catchment area of care center | 59 children with partial motor seizures | 50 with CT–scan | According to [30] | Partial motor seizures: characterized by convulsive activity involving any part of the body (hands, face, legs, etc.,) [41] | Seizures | Proportion of NCC among children with partial motor seizures | Census | Yes |
| India,  2004–05 | [59] (English) | Cross–sectional | 1,640 residents of a rural pig farming community of Mohanlalganj , Lucknow,Uttar Pradesh | 95 people with active epilepsy | 94 who accepted MRI | According to [4, 54] | AE: according to [32] | AE (100%, defined as one of ≥ 2 seizures within 5 years) | Prevalence of NCC among people with active epilepsy | 30–cluster sampling [60] | Yes |
| India,  2006–12 | [61] (English) | Cross–sectional | First to fifth grader students of govt primary school adopted by NICE foundation, Hyderabad | 30 children (age: 5–15 years) with new–onset seizures during the study period & diagnosed as epilepsy | 30 children with epilepsy who had CT–scan | According to [4] | Epilepsy: according to [20]. Seizures were classified according to [62] | Epilepsy | Prevalence of single calcific–NCC among school going children (1^st^–5^th^ grader) with epilepsy | School cluster/ Census | Yes |
| India,  2006–12 | [63] (English) | Cross–sectional | 7,408 Students of government primary schools, Hyderabad, | 58 children (age: 5–15 years) with new–onset seizures who had CT | 58 | According to [4] | Seizures were categorized according to [64] | Seizures | Prevalence of degenerative phase of NCC among school going children with new–onset seizures | School cluster/ Census | Yes |
| India,  2009 | [65] (English) | Case series | People from catchment area of the hospital | 87 consecutive patients with complaint of seizures | 79 patients with epilepsy | According to [17, 66] | Assumed epilepsy was diagnosed by neurologist | Epilepsy | Proportion of NCC among patients with epilepsy | Census | No |
| India,  2010–11 | [67] (English) | Cross–sectional | 15,750 people of a slum community of Ludhiana, Punjab | 114 people with active epilepsy | 93 who had MRI | According to [4] | Epilepsy/AE: according to [32] | AE (100%, defined as one of ≥ 2 seizures within 5 years) | Prevalence of NCC among people with active epilepsy | Census/door–to–door survey | Yes |
| India,  2010–11 | [67] (English) | Cross–sectional | 15,750 people of a slum community of Ludhiana, Punjab | 27 seropositive (EITB positive) people with active epilepsy | 26 who had MRI | According to [4] | Epilepsy/AE: according to [32] | AE (100%, defined as one of ≥ 2 seizures within 5 years) | Prevalence of NCC among seropositive people with active epilepsy | Census/door–to–door survey | Yes |
| India,  2011–12 | [68] (English) | Case series | Children from catchment area of the hospital | 150 children with focal seizures | 143 who underwent brain imaging | By a pediatric neurologist according to [69] | Focal seizures: defined according to [21] | Seizures | Proportion of NCC among children with focal seizures | Census | Yes |
| India,  2013–14 | [70] (English) | Case series | People attending the Sree Balaji Medical college and Hospital | 50 hospitalized adult patients (age: 18–70 years) with new onset focal seizures | 50 who had MRI/CT–scan | Assumed NCC was diagnosed by neurologists | Seizures were categorized according to the classification proposed by ILAE 2005–09 | Seizures | Proportion of NCC among adult patients with new onset focal seizures | Census | No |
| India,  2013–16 | [71] (English) | Case series | People attending the SCB Medical College and Hospital, Cuttack, Odisha | 300 patients with newly diagnosed epilepsy (NDE) | 300 who had MRI/CT–scan | Assumed NCC was diagnosed by neurologists | Epilepsy: according to [20]. NDE: diagnosed first time during the study period [72] | Epilepsy | Proportion of NCC among newly diagnosed epilepsy | Census | No |
| India,  2015–16 | [73] (English) | Case series | Children of catchment area of a tertiary hospital of Uttar Pradesh | 115 children (age: ≤ 14 years) with first unprovoked seizures (FUS) ^3^ | 115 who had MRI/CT–scan | NCC was diagnosed by neurologists | FUS is a type of seizure with no obvious precipitating cause, although improvement of diagnostic techniques has increased the probability of finding the unknown causes. Types of seizures defined according to [21] | Seizures | Proportion of NCC among children with first unprovoked seizures | Census | No |
| India,  2016 | [74] (English) | Case series | People attending the Maharani Laxmibai Medical College, Jhansi | 176 patients with epilepsy attending epilepsy clinic | 176 who had CT–scan | Assumed NCC was diagnosed by neurologists | Seizures were classified according to [21] | Epilepsy | Proportion of NCC among patients with epilepsy | Census | No |
| India,  2017–18 | [75] (English) | Case series | Children attending the TMMRC Moradabad. | 100 consecutive children (age: 1.5–18 years) with seizures | 100 who had MRI | According to [2] | Seizures were defined according to [76] | Seizures | Proportion of NCC among children with seizures | Census | Yes |
| India,  2017–18 | [77] (English) | Cross–sectional | 59,509 screened people of urban and periurban rural areas of Ludhiana, Panjab | 240 PWE | 200 who had MRI | According to [10] | Epilepsy: defined according to [20, 62] | Epilepsy | Prevalence of NCC among people with epilepsy | Cluster sampling/door–to–door survey | Yes |
| India,  2017–20 | [78] (English) | Case series | Catchment population of a referral center in Northeast India | 630 consecutive patients with epilepsy who had MRI | 630 with MRI | Assumed NCC was diagnosed by neurologists / radiologists | Epilepsy was defined according to [32] | Epilepsy | Proportion of NCC among patients with epilepsy | Census | No |
| India,  2018–19 | [79] (English) | Case series | Children attending the Bhagat Phool Singh Government Medical College for Women, Sonepat district, Haryana | 92 consecutive children (age: 1–15 years) admitted with a first–onset seizures | 79 who had MRI/CT–scan | According to [4] | Assumed seizures were diagnosed by pediatric physician | Seizures | Proportion of NCC among children with a first–onset seizures | Census | No |
| Italy,  2001–16 | [80] (English) | Cross–sectional | IAC who underwent serological screening in two pediatric reference centers | 7 seronegative IAC (age: ≤ 16 years) with epilepsy who had MRI | 7 with MRI | According to [10] | Assumed epilepsy was diagnosed by neuro–expert | Epilepsy | Prevalence of NCC among internationally adopted (seronegative) children with epilepsy | Census | No |
| Malawi, 2012–13 | [81] (English) | Cross–sectional | 69,595 surveyed residents of Balaka district of southern Malawi | 365 PWE (age: > 6 years) with full clinical information | 127 PWE^1^ (age: > 6 years) who accepted CT–scan | According to [10] | Epilepsy: ≥ 2 unprovoked (or reflex) seizures occurring more than 24 hours apart. | AE (31.2%, defined as presence of epileptic seizures in past 5 years) | Prevalence of NCC among people with epilepsy | Census/door–to–door survey | Yes |
| Mexico,  NA–NA | [82] (English) | Case series | People from catchment area of the hospital | 100 consecutive adult (age: ≥ 25 years) patients with late–onset epilepsy | 100 who had CT–scan | According to [15] | Late–onset epilepsy: seizures occurred after 25 years of age. Seizures were classified according to [5] | Late–onset epilepsy | Proportion of NCC among adult patients with late–onset epilepsy | Census | Yes |
| Mexico  NA–NA | [83] (English) | Case series | Children attending the Hospital Infantil de México | 41 children (age: 3–18 years) with seizures | 38 who had MRI/CT–scan | According to [4, 84] | Assumed seizures were diagnosed by neurologists | Seizures | Proportion of NCC among children with seizures | Census | No |
| Mexico, 1989–90 | [85] (Spanish) | Case series | Children attending the National Medical Center Siglo XXI, Mexican Institute of Social Security | 118 children with epilepsy who had CT–scan | 118 | CT lesions were classified according to [15] | Seizures were classified according to [5] | Epilepsy | Proportion of NCC among children with epilepsy** | Census | Yes |
| Mexico, 1989–97 | [86] (Spanish) | Case series | People from catchment area of a Mexican hospital | 130 adult (age: ≥ 20 years) patients with late–onset epilepsy | 130 who had MRI/CT–scan | Assumed NCC was diagnosed by neuro–expert | Late–onset epilepsy: seizures occur after 20 years of age. Epileptic seizures were classified according to [5] | Late–onset epilepsy | Proportion of NCC among adult patients with late–onset epilepsy | Census | No |
| Mexico,  2000 | [87] (Spanish) | Case series | Residents of Guadalajara, Tampico, Cuernavaca, Tijuana, Monterrey and Federal District | 455 adult (age: ≥ 20 years) patients with late–onset epilepsy who visited ten neurology centers | 455 who had CT–scan | (1) Cystic lesion with or without contrast medium enhancement, with or without perilesional edema. or (2) a parenchymal calcification or several calcifications of > 0.5 centimeters, without perilesional edema. | Late–onset epilepsy: seizures occur after 20 years of age. Seizures were classified according to [5] | Late–onset epilepsy | Proportion of NCC among adult patients with late–onset epilepsy | Census | Yes |
| Mozambique, 2007 | [88] (English) | Cross–sectional | 1723 People screened from 18 villages of Angónia district, Tete province | 151 confirmed PWE who had CT–scan | 151 | According to [2, 4] | Epilepsy defined according to [32, 89] | Epilepsy | Prevalence of NCC among people with epilepsy | Census | Yes |
| Namibia, 2012–14 | [90] (English) | Case series | People attending the Intermediate Hospital Oshakati Hospital | 221 patients with first time generalized seizures | 177* who had CT–scan | According to [4] | An individual presenting with an unprovoked seizure. Assumed seizures were diagnosed by expert | Seizures | Proportion of NCC among patients with first–time generalized seizures | Census | Yes |
| Nepal,  2014–16 | [91] (English) | Case series | Children of catchment area of a medical college hospital | 168 hospitalized children (age: ≤ 16 years) with seizures | 168 with CT–scan | According to [2] | Seizure was defined as a transient occurrence of signs and/or symptoms resulting from abnormal excessive or synchronous neuronal activity in the brain. | Seizures | Proportion of NCC among children with seizures | Census | Yes |
| Nepal,  2017–18 | [92] (English) | Case series | People attending the B & C Medical College | 103 hospitalized patients with seizures | 103 with MRI/CT–scan | According to [2] | Assumed seizures were diagnosed by expert | Seizures | Proportion of NCC among people with seizures | Census | No |
| Peru,  NA–NA | [93] (English) | Cross–sectional | 576 people eligible for ring screening (living in 31 ring) of the village of Surpampa, Piura | 16 PWE | 12 who accepted CT–scan | Parenchymal calcification or cystic lesion was considered NCC. Diagnosed by physician | Seizures & epilepsy were diagnosed according to Fisher, 2014 #77} | AE (31.3%, defined as 1 seizures in last 5 years) | Prevalence of NCC among people with epilepsy | Cluster sampling | Yes |
| Peru,  1998–99 | [94] (Spanish) | Case series | Children from catchment area of The Cayetano Heredia National Hospital | 54 children with partial seizures attended to outpatient clinic & emergency department | 54 who had CT–scan | According to [4] | Epileptic seizures were classified according to [5] | Seizures | Proportion of NCC among children with partial seizures | Census | Yes |
| Peru,  1999–2000 | [95] (English) |  | 903 Resident of Matapalo, in the Zarumilla River Valley, near the Peru–Ecuador border | 43 people with confirmed seizures | 39 who had CT–scan | According to [66] | Seizures were diagnosed according to [96] | Active & inactive epilepsy, single seizure | Prevalence of NCC among people with epileptic seizures | Census/door–to–door survey | Yes |
| Peru,  1999–05 | [97] (English) | Cross–sectional | 817 residents of Matapalo district included in a cohort | 8 individuals with epileptic seizures (6 PWE) | 8* with MRI/CT–scan | Assumed the NCC was diagnosed by neurologists | Epilepsy/Epileptic seizures were defined according to [32] | Seizures (6 had epilepsy) | Prevalence of NCC among people with epileptic seizures | Census/door–to–door survey | No |
| Peru,  2006–07 | [98] (English) | Cross–sectional | 17,450 people surveyed from 58 communities of left and right bank of Tumbes River | 301 PWE | 282 PWE^1^ with CT–scan | According to [99, 100] | Epilepsy was defined according to [35, 89] | Epilepsy | Prevalence of NCC among people with epilepsy | Census/door–to–door survey | Yes |
| Peru,  2006–07 | [98] (English) | Cross–sectional | 17,450 people surveyed from 58 communities of left and right bank of Tumbes River | 279 PWE who had EITB & CT–scan | 115 seropositive individuals with CT–scan | According to [99, 100] | Epilepsy was defined according to [35, 89] | Epilepsy | Prevalence of NCC among seropositive (EITB positive) people with epilepsy | Census/door–to–door survey | Yes |
| Peru,  2006–07 | [98] (English) | Cross–sectional | 17,450 people surveyed from 58 communities of left and right bank of Tumbes River | 279 PWE who had EITB & CT–scan | 164 seronegative individuals | According to [99, 100] | Epilepsy was defined according to [23, 89] | Epilepsy | Prevalence of NCC among seronegative (EITB negative) people with epilepsy | Census/door–to–door survey | Yes |
| Qatar,  2012–13 | [101] (English) | Case series | Children of catchment area of the pediatric emergency center | 96 children (age: < 14 years) with first non–febrile seizures | 96 who had MRI/CT–scan | NCC was diagnosed by neurologist | Seizures were diagnosed according to [21] | Seizures | Proportion of NCC among children with first non–febrile seizures** | Census | No |
| South Africa,  2004–05 | [102] (English) | Case series | People from catchment area of St. Elizabeth’s Hospital in Lusikisiki, Eastern Cape Province | 281 individuals (age ≥ 5 years) with epilepsy who attended epilepsy clinic | 92 randomly selected patients who had CT–scan | Either evidence of visible cysticerci, colloidal cysts or presence of cerebral calcifications | Classification of seizures according to [5, 35]; Epilepsy: ≥ 2 seizures separated by at least 24 hours. | Epilepsy | Proportion of NCC among people with epilepsy | Random sampling | Yes |
| South Africa, 2016–18 | [103] (English) | Case series | People of catchment area of a tertiary hospital | 531 adult (age: > 18 years) patients with new–onset of seizures | 531 whose CT–scan results available in hospital picture archiving & communication system | NCC was diagnosed by radiologists | Seizures were defined according to [104] | Seizures | Proportion of NCC among adults (age: > 18 years) with new–onset seizures** | Census | No |
| Thailand, 2003–05 | [105] (English) | Case series | People attending the epilepsy clinic of Srinagarind Hospital | 180 patients (age: > 15 years) with epilepsy who had MRI/CT–scan | 180 | NCC was diagnosed by neuroradiologist | Epilepsy was diagnosed by a neurologist. Seizures were classified according to [5] | Epilepsy | Proportion of NCC among patients with epilepsy | Census | No |
| Uganda, 2009–12 | [106]ᵇ (English) | Cross–sectional | 38,303 surveyed people of Moyo, Gulu and Adjumani districts | 300 people with active epilepsy who had CT–scan | 300 | According to [2, 4] | Epilepsy: according to [89]. Active epilepsy: who had seizures within last 12 months [107] | AE (100%; defined as seizures within 12 months) | Prevalence of NCC among people with active epilepsy | Village cluster/ door–to–door survey | Yes |
| United Republic of Tanzania,  2006 | [108] (English) | Case series | People of catchment area of Haydom Lutheran Hospital, Mbulu district | 219 people (age: > 10 year) with epilepsy | 212 PWE who accepted CT–scan | According to [4] | Epilepsy: ≥ 2 afebrile seizures unrelated to acute metabolic disorders or withdrawal of drugs or alcohol | Epilepsy | Proportion of NCC among people with epilepsy | Non–probability | Yes |
| United Republic of Tanzania, 2009 | [109] (English) | Cross–sectional | 161,119 people of Hai district, Tanzania | 291 PWE | 200 for whom CT–scan available | According to [4] | Epilepsies/seizures were classified according to [21] | Epilepsy | Prevalence of NCC among people with epilepsy | Census/door–to–door survey | Yes |
| United Republic of Tanzania, 2009 | [110] (English) | Cross–sectional | 830 screened people of Mbozi district, Mbeya Region | 427 seropositive (Ag–ELISA positive) individuals | 28* with epileptic seizures who had CT–scan | NCC was diagnosed by radiologist | Epilepsy: ≥ 2 unprovoked epileptic seizures | Seizures | Prevalence of NCC among seropositive (Ag–ELISA positive) people with epileptic seizures | Cluster sampling (Household)/ door–to–door survey | No |
| United Republic of Tanzania, 2009 | [111] (English) | Cross–sectional | 38,523 screened children of Hai district | 112 children (age: 6–14 year) with active epilepsy | 90 who had CT | NCC was diagnosed by pediatric neuroradiologist | Active epilepsy was defined according to [32] | AE (100%, defined as epilepsy in last 5 years) | Prevalence of NCC among children with active epilepsy | Census/door–to–door survey | No |
| United Republic of Tanzania, 2012–13 | [112] (English) | Case series | People of Kinondoni municipality, Dar es Salaam who had access to six government health centers | 600 PWE diagnosed based on review of health record. | 302 randomly selected PWE who underwent CT–scan | According to [10] | Epilepsy was defined according to [20] | Epilepsy | Proportion of NCC among people with epilepsy | Random sampling | Yes |
| United Republic of Tanzania, 2018–20 | [113] (English) | Cross sectional | People of catchment area of hospitals of three districts – Ifisi, Tukuyu,Vwawa | 58 people with epileptic seizures who were positive to POC–test^2^ & underwent CT–scan | 58 | According to [10] | Seizures were classified according to [20] | Seizures | Proportion of NCC among POC–test^2^ positive patients with epileptic seizures | Census | Yes |
| United Republic of Tanzania, 2018–20 | [113] (English) | Cross sectional | People of catchment area of hospitals of three districts – Ifisi, Tukuyu,Vwawa | 31 people with epileptic seizures who were negative to POC–test^2^ & underwent CT–scan | 31 | According to [10] | Seizures were classified according to [20] | Seizures | Proportion of NCC among POC–test^2^ negative patients with epileptic seizures | Census | Yes |
|  |  |  |  |  |  |  |  |  |  |  |  |
| USA,  1996–98 | [114] (English) | Case series | People from catchment area of 11 emergency departments (EMERGEncy  ID NET) | 1,801 patients who visited emergency department with known or suspected seizures | 1,801 who underwent brain imaging (CT/MRI) | According to [17] | Assumed seizures were diagnosed by neurology expert | Seizures | Proportion of NCC among (adult) patients with seizures | Census | No |
| USA,  2013 | [115] (English) | Case series | Catchment population of a community–based neurology clinic, Los Angeles | 160 adult (age: > 18 year) patients with epilepsy | 160 who had neuroimaging results in medical report† | According to [116] | Medical chart review; Assumed seizures were diagnosed by neurology expert | Epilepsy | Percentage of NCC among adult (age: > 18 year) patient with epilepsy** $ | Census | No |
|  |  |  |  |  |  |  |  |  |  |  |  |

*Age group and/or gender of the study population (study subjects who had brain imaging) is unknown;

**Retrospective review of medical records/case notes/autopsy protocols/death certificates;

$ Diagnosis of NCC or neurological manifestation involve the search of hospital’s/local/national health database using International Classification (ICD) code: ICD–9 or ICD–10;

†: Types of imaging used for NCC diagnosis was not specified in the article;

‡: Study was done after the implementation of epilepsy control program;

^1^Study population includes both seropositive and seronegative individuals with epilepsy, but brain imaging of the studied individuals was not determined based on their serostatus;

^2^POC–test: a novel antibody–detecting lateral–flow point–of–care test for the diagnosis of *T*. *solium* taeniosis (targeting rES33 protein) and (neuro)cysticercosis (targeting rT24H protein);

^3^Excluded subjects with simple febrile seizures, acute situational seizures due to toxin, infection, or trauma, known epileptic disorder, and history of seizures;

ªChildren with hydrocephalus due to tumor were excluded from the study;

ᵇThis article is a thesis (not peer–reviewed articles);

^c^See section “Operational definition of terms used in this study” of the main article for detail.

*AE* active epilepsy; *Ag* antigen; *CSF* cerebrospinal fluid; *CT* computed tomography; *EEG* electroencephalogram ; *EITB* enzyme–linked immunoelectrotransfer blot techniques; *ELISA* enzyme–linked immunosorbent assay; *h* hours; *HIV* human immunodeficiency virus; *IAC* internationally adopted children; *ICD* International Classification of Disease code; *MRI* magnetic resonance imaging; *NA* not available; *NCC* neurocysticercosis; *NDE* newly diagnosed epilepsy; *POC* point of care; *PWE* people with epilepsy;

# Table S5. Summary of study–specific posterior distributions of the frequency of NCC among people with epileptic seizures

| **Country, year(s) of study** | **Study** | **Measure of frequency** | **Median % (95% *BCI*)** |
| --- | --- | --- | --- |
| Bhutan,  2014–15 | [1] | Percentage of NCC among people with epilepsy who visited a national tertiary referral center & had brain MRI^1^ | 13.4 (9.3–18.7) |
| Bolivia,  1994 | [3] | Prevalence of NCC among patients with epilepsy living in rural communities & who had brain CT–scan | 28.0 (20.1–37.0) |
| Brazil,  NA–NA | [9] | Percentage of calcified NCC among patients (age: > 15 years) with epilepsy who participated in a cohort followed in a referral hospital & had brain MRI/CT–scan | 6.5 (4.8–8.5) |
| Brazil,  NA–NA | [6] | Percentage of NCC among patients (age: ≥ 10 years) with chronic epilepsy who were evaluated and followed for three years in a service neurology & had brain CT–scan | 27.3 (21.5–33.5) |
| Brazil,  1978–90 | [13] | Percentage of NCC among patients with epileptic seizures who attended a brain imaging center & had brain CT scan | 23.6 (21.1–26.3) |
| Brazil,  1995–96 | [14] | Percentage of NCC among patients with epileptic seizures who were hospitalized in a regional hospital & had brain CT–scan** | 24.8 (14.9–36.7) |
| Brazil,  2005 | [16] | Percentage of intracranial calcification suggestive of NCC among patients with seizures (although author mentioned the manifestation as “epilepsy” based on medical chart–review) who attended to two university hospitals & had brain CT–scan** | 17.1 (10.7–25.1) |
| Brazil,  2009–11 | [18] | Percentage of NCC among patients with epilepsy who were followed in a specialized epilepsy clinic & had brain MRI/CT–scan* | 15.0 (12.2–18.5) |
| Brazil,  2013 | [19] | Percentage of NCC among people with epilepsy seen in epilepsy clinics of a hospital & had brain MRI/CT–scan | 15.1 (11.2–19.7) |
| Burkina Faso,  2007 | [22] | Prevalence of NCC among people with epilepsy living in village communities & had brain CT–scan | 28.4 (18.6–39.6) |
| Cameroon,  1999–05 | [24] | Percentage of NCC among people (age: > 15 years) with epilepsy who were followed for seven years in neurology department of a hospital & had complete medical files**† | 7.4 (3.0–14.6) |
| Colombia,  1995 | [25] | Percentage of NCC among patients with epilepsy who attended outpatient–clinics & had brain CT–scan | 14.0 (11.3–17.2) |
| Côte d'Ivoire,  1994–96 | [26] | Percentage of NCC among people with epileptic seizures who had brain CT–scan in a polyclinic** | 2.7 (1.1–5.6) |
| Côte d'Ivoire,  2015–17 | [28] | Percentage of NCC among individuals with epileptic seizures who visited the neurology units of three urban hospitals & had brain CT–scan results in their medical records | 3.2 (0.9–7.5) |
| Ecuador,  1986–90 | [33] | Percentage of NCC among adult patients with late–onset (seizures occurred after 25 years of age) of epilepsy who attended a hospital & had brain CT–scan | 38.3 (32.2–44.8) |
| Ecuador,  1990–09 | [117] | Percentage of NCC among adult patients with late–onset (recurrent seizures starting after the age of 20 years) epilepsy who visited neurology unit of a private hospital & had brain MRI/CT–scan | 27.9 (23.9–32.2) |
| Ecuador,  1992–92 | [29] | Prevalence of NCC among people with epilepsy living in a community & had brain CT–scan | 53.5 (34.9–71.1) |
| Ecuador,  2003 | [34] | Prevalence of NCC among people with epilepsy living in a community & had brain CT–scan | 27.7 (11.3–49.5) |
| Ecuador,  2012–15 | [37] | Prevalence of NCC among adult people (age: ≥ 20 years) with epilepsy living in a rural community & had brain CT–scan | 23.9 (12.6–38.8) |
| Ecuador,  2012–15 | [37] | Prevalence of NCC among adult people (age: ≥ 20 years) with single seizure living in a rural community & had brain CT–scan | 31.9 (8.3–64.9) |
| Honduras,  1997–05 | [40] | Prevalence of NCC among people with active epilepsy living in village communities & had brain CT–scan‡ | 16.6 (6.9–31.2) |
| Honduras,  1997 | [39] | Prevalence of NCC among people with active epilepsy living in a community & had brain CT–scan | 36.9 (27.5–47.3) |
| India,  NA–NA | [47] | Prevalence of NCC among people (age: ≥ 5 years) with active epilepsy in a rural tea garden community & had brain CT–scan | 42.2 (28.9–56.8) |
| India,  NA–NA | [50] | Percentage of NCC among patients with new–onset seizures & had brain MRI | 6.8 (3.3–12.0) |
| India,  NA–NA | [51] | Prevalence of NCC among people with active epilepsy living in village communities & had brain MRI/CT–scan | 4.06 (22.9–59.6) |
| India,  NA–NA | [52] | Prevalence of NCC among people with active epilepsy living in village communities & had brain CT–scan | 34.9 (27.5–42.7) |
| India,  NA–NA | [46] | Percentage of NCC among children with partial seizures & had brain CT–scan ** | 13.5 (7.6–20.8) |
| India,  NA–NA | [53] | Prevalence of NCC people with epilepsy living in a pig farming community & had brain MRI* | 48.3 (36.0–60.4) |
| India,  1995–01 | [43] | Percentage of NCC among patients with single seizure who attended an outpatient service of a hospital & brain CT–scan | 62.2 (56.7–67.5) |
| India,  1995–01 | [43] | Percentage of NCC among patients with recurrent acute symptomatic seizures who attended an outpatient service of a hospital & brain CT–scan | 91.7 (87.1–95.1) |
| India,  1995–01 | [43] | Percentage of NCC among patients with epilepsy who attended an outpatient service of a hospital & brain CT–scan | 1.9(0.9–3.4) |
| India,  1992–93 | [41] | Percentage of NCC among children (age: ≤ 15 years) with simple partial seizures who attended a health center & had brain CT–scan | 10.4 (6.6–15.2) |
| India,  1995–01 | [55] | Percentage of NCC among patients with new–onset seizures (both acute symptomatic and unprovoked) seen in the neurology unit of a hospital & had brain MRI/CT–scan | 59.7 (54.1–65.1) |
| India,  1995–99 | [42] | Percentage of NCC among children with partial seizures who attended a health institute & had brain CT–scan | 2.4 (0.7–5.7) |
| India,  2001 | [57] | Prevalence of calcified NCC among people with epilepsy living in village communities & had brain CT–scan | 19.3 (15.6–23.5) |
| India,  2001–02 | [58] | Percentage of NCC among children (age: 1 month–12 years) with partial motor seizures who attended a tertiary care center & had brain CT–scan | 7.1 (2.2–16.0) |
| India,  2003–04 | [44] | Prevalence of NCC among people with active epilepsy who were living in an urban community & had brain CT–scan | 28.5 (21.8–35.8) |
| India,  2004–05 | [59] | Prevalence of NCC among people (study population was selected following 30–cluster sampling approach) with active epilepsy living in a pig farming community & had brain MRI | 46.9 (37.0–56.9) |
| India,  2006–12 | [61] | Percentage of single calcific–NCC among school going (1st to 5th grade) children (age: 5–15 years) with epilepsy who were referred to a hospital & had brain CT–scan | 18.1 (7.5–33.4) |
| India,  2006–12 | [63] | Percentage of degenerative phase of NCC among school–going children (age: 5–15 years) with new–onset seizures who were referred to a hospital & had brain CT–scan | 33.1 (22.1–45.5) |
| India,  2009 | [65] | Percentage of NCC among patients with epilepsy attended to neurology OPD of a tertiary hospital & had brain CT–scan | 40.6 (30.1–51.8) |
| India,  2010–11 | [67] | Prevalence of NCC among people with active epilepsy living in a slum community & had brain MRI | 20.9 (13.4–29.8) |
| India,  2010–11 | [67] | Prevalence of NCC among seropositive (EITB positive) people with active epilepsy living in a slum community & had brain MRI | 30.9 (16.3–50.6) |
| India,  2011–12 | [68] | Percentage of NCC among children with focal seizures who attended a tertiary hospital & had brain CT–scan | 40.0 (32.2–48.0) |
| India,  2013–14 | [70] | Percentage of NCC among adult patients (age: 18–70 years) with new onset focal seizures admitted to a medical college hospital & had brain MRI/CT–scan | 20.8 (11.2–32.8) |
| India,  2013–16 | [71] | Percentage of NCC among newly diagnosed epilepsy attended to the neurology unit of a tertiary care hospital & had brain MRI/CT–scan | 19.7 (15.1–25.1) |
| India,  2015–16 | [73] | Percentage of NCC among children (age: 0.5–14 years) with first unprovoked seizures^3^ | 14.3 (8.9–21.4) |
| India,  2016 | [74] | Percentage of NCC among people with epilepsy attended to epilepsy clinic of a tertiary hospital & had brain CT–scan | 14.5 (9.8–20.3) |
| India,  2017–18 | [75] | Percentage of NCC among children (age: 1.5–18 years) with seizures attended to a tertiary hospital & had brain MRI | 38.1 (29.2–47.8) |
| India,  2017–18 | [77] | Prevalence of NCC among people with epilepsy living in urban and peri–urban rural communities & had brain MRI | 14.2 (9.8–19.4) |
| India,  2017–20 | [78] | Percentage of NCC among patients with epilepsy who had MRI in the radiology unit of a referral center | 12.6 (10.2–15.4) |
| India,  2018–19 | [79] | Percentage of NCC among children (age: 1–15 years) with first–onset seizures who were admitted to the emergency room of a government medical college & had brain MRI/CT–scan | 54.4 (43.4–65.0) |
| Italy,  2001–16 | [80] | Percentage of NCC among internationally adopted children (age: ≤ 16 years) with epilepsy evaluated in two pediatric reference centers | 79.9 (47.2–96.8) |
| Malawi,  2012–13 | [81] | Prevalence of NCC among people with epilepsy living in a (district) community & had brain CT–scan^1^ | 4.4 (1.8–8.9) |
| Mexico,  NA–NA | [83] | Percentage of NCC among children (age: 3–18 years) with seizures who were treated at the neurology unit of a hospital & had brain MRI/CT–scan | 6.8 (1.6–17.0) |
| Mexico,  NA–NA | [82] | Percentage of NCC among adult patients with late–onset (seizures occurred after 25 years of age) epilepsy who attended the epilepsy clinic of national neurology institute & had brain CT–scan | 50.0 (40.6–59.7) |
| Mexico,  1989–90 | [85] | Percentage of NCC among children with epilepsy who were referred to the neurology service of a pediatric hospital & had brain CT–scan** | 10.6 (6.0–17.0) |
| Mexico,  1989–97 | [86] | Percentage of NCC among adult patients with late–onset (seizures occurred after 20 years of age) epilepsy who attended a hospital & had brain MRI/CT–scan | 28.7 (21.5–36.7) |
| Mexico,  2000 | [87] | Percentage of NCC among adult people with late–onset (epilepsy starting after the age of 20 years) epilepsy who visited 10 neurology centers & had brain CT–scan | 21.2 (17.6–25.0) |
| Mozambique,  2007 | [88] | Prevalence of NCC among people with epilepsy (lifetime epilepsy) living in rural communities & had brain CT–scan | 56.2 (48.1–63.9) |
| Namibia,  2012–14 | [90] | Percentage of NCC among patients (age: ≥ 12 years) with first–time generalized seizures presented to a referral hospital & had brain CT–scan* | 54.2 (46.8–61.6) |
| Nepal,  2014–16 | [91] | Percentage of NCC among children (age: 0–16 years) with seizures who were admitted to the neurology department of a medical college & had brain CT–scan | 43.0 (35.8–50.5) |
| Nepal,  2017–18 | [92] | Percentage of NCC among people with seizures who were admitted to a hospital & had brain MRI/CT–scan | 28.5 (20.4–37.9) |
| Peru,  NA–NA | [93] | Prevalence of NCC among people with epilepsy living in a rural community & had brain CT–scan | 42.7 (19.5–68.0) |
| Peru,  1998–99 | [94] | Percentage of NCC among children (age: 2–14) with partial seizures who attended a national hospital & had brain CT–scan | 26.3 (15.7–39.2) |
| Peru,  1999–2000 | [95] | Prevalence of NCC among people with epileptic seizures (single seizure/epilepsy) living in a community & had brain CT–scan | 38.9 (24.8–54.4) |
| Peru,  1999–05 | [97] | Prevalence of NCC among people with epileptic seizures (new–onset epileptic seizures) living in a rural community & had brain MRI/CT–scan* | 39.3 (13.5–69.6) |
| Peru,  2006–07 | [98] | Prevalence of NCC among seropositive (EITB positive) people (age: > 2 years) with epilepsy living in communities & had brain CT–scan | 53.9 (44.8–62.7) |
| Peru,  2006–07 | [98] | Prevalence of NCC among seronegative (EITB negative) people (age: > 2 years) with epilepsy living in communities & had brain CT–scan | 28.9 (22.3–36.2) |
| Peru,  2006–07 | [98] | Prevalence of NCC among people (age: > 2 years) with epilepsy living in communities & had brain CT–scan^1^ | 38.7 (33.0–44.4) |
| Qatar,  2012–13 | [101] | Percentage of NCC among children (age: < 14 years) with first non–febrile seizures attended to a pediatric emergency center & had brain MRI/CT–scan | 1.7 (0.3–5.6) |
| South Africa,  2004–05 | [102] | Percentage of NCC among patients (age: ≥ 5 years) with epilepsy who attended a rural epilepsy clinic & had CT–scan | 37.1 (27.7–47.1) |
| South Africa,  2016–18 | [103] | Percentage of NCC among adults (age: > 18 years) with new–onset seizures attended to a tertiary hospital & had medical record with brain CT–scan results in hospital picture archiving & communication system** | 2.8 (1.6–4.4) |
| Thailand,  2003–05 | [105] | Percentage of NCC among patients (age: > 15 years) with epilepsy who underwent brain MRI/CT–scan in an adult epilepsy clinic | 5.3 (2.6–9.3) |
| Uganda,  2009–12 | [106] | Prevalence of NCC among people with active epilepsy living in communities & had brain CT–scan^4^ | 13.5 (10.1–17.7) |
| United Republic of Tanzania,  2006 | [108] | Percentage of NCC among patients with epilepsy who attended a rural hospital & had brain CT–scan | 18.1 (13.4–23.8) |
| United Republic of Tanzania,  2009 | [109] | Prevalence of NCC among people (age: ≥ 15 years) with epilepsy living in a (district) community & had brain CT–scan | 4.3 (2.1–7.7) |
| United Republic of Tanzania,  2009 | [110] | Prevalence of NCC among seropositive (Ag–ELISA positive) people with epilepsy living in a rural community & had brain CT–scan* | 97.7 (88.2–99.9) |
| United Republic of Tanzania,  2009 | [111] | Prevalence of NCC among children (age: 6–14 year) with active epilepsy living in a (district) community & had brain CT–scan | 2.9 (0.7–7.8) |
| United Republic of Tanzania,  2012–13 | [112] | Percentage of NCC among people with epilepsy who went to six government health centers & had brain CT–scan | 2.2 (0.9–4.3) |
| United Republic of Tanzania,  2018–20 | [113] | Percentage of NCC among POC–test^2^ positive patients with epileptic seizures attended the Mental Health Clinics of three district hospitals & had brain CT–scan | 77.0 (65.4–86.6) ^§^ |
| United Republic of Tanzania,  2018–20 | [113] | Percentage of NCC among POC–test^2^ negative patients with epileptic seizures attended the Mental Health Clinics of three district hospitals & had brain CT–scan | 26.8 (13.7–43.9) ^§^ |
| USA,  1996–98 | [114] | Proportion of NCC among patients with seizures who visited emergency department & had brain CT–scan | 2.1 (1.6–2.9) |
| USA,  2013 | [115] | Percentage of NCC among adult (age: > 18 year) patients with epilepsy presented to a community–based neurology clinic & had neuroimaging result**$† | 39.5 (32.1–47.0) |

*Age group and/or sex of the study population (study subjects who had brain imaging) is unknown.

**Retrospective review of medical records/autopsy protocols/death certificate.

$ Diagnosis of NCC or neurological manifestation involve the search of hospital’s/local/national health database using International Classification of Disease (ICD) code: ICD–9 or ICD–10;

†: Types of imaging used for NCC diagnosis were not specified in the article.

‡: Study was done after the implementation of an epilepsy control program.

§: Authors reported overall frequency of NCC among PWE at 35% (95%*CI*: 31–39%) after weighting for the distribution of the results of the POC–test^2^;

^1^Study population includes both seropositive and seronegative individuals with epilepsy.

^2^POC–test: a novel antibody–detecting lateral–flow point–of–care test for the diagnosis of *T*. *solium* taeniosis (targeting rES33 protein) and (neuro)cysticercosis (targeting rT24H protein);

^3^Excluded subjects with simple febrile seizures, acute situational seizures due to toxin, infection, or trauma, known epileptic disorder, and history of seizures.

^4^This is a thesis (not peer–reviewed articles).

*Ag* antigen; *BCI* Bayesian credible interval; *CT* computed tomography; *EEG* electroencephalogram ; *EITB* enzyme–linked immunoelectrotransfer blot; *ELISA* enzyme–linked immunosorbent assay; *IAC* internationally adopted children; *ICD* International Classification of Disease code; *MRI* magnetic resonance imaging; *NA* not available; *NCC* neurocysticercosis; *POC* point-of-care; *PWE* people with epilepsy; *%* percentage.

# Table S6. Summary of meta-analysis results stratified by age group (children and adults) and sex (male and female).

| Variables | Number of studies  *n* | Proportion of NCC  % (95% *BCI*) | Heterogeneity  *τ* (95% *BCI*)^b^ |
| --- | --- | --- | --- |
| Estimates only using studies *with* definitions of NCC and epileptic seizures or epilepsy | | | |
| *Age groups of studied populations^a^* | | | |
| Children | 17 | 23.4 (16.7–31.7) | 0.8 (0.6–1.2) |
| Adult | 14 | 26.8 (18.3–37.6) | 0.9 (0.6–1.4) |
| *Biological sex of studied populations* | | | |
| Male | 8 | 23.2 (9.0–49.4) | 1.7 (0.9–3.1) |
| Female | 8 | 24.3 (9.8–50.6) | 1.6 (0.9–2.8) |
| Estimates only using studies *with* definitions of NCC and epilepsy | | | |
| *Age groups of studied populations^a^* | | | |
| Children | 9 | 20.0 (13.3–29.5) | 0.7 (0.3–1.2) |
| Adult | 12 | 23.3 (15.7–33.2) | 0.9 (0.5–1.3) |
| *Biological sex of studied populations* | | | |
| Male | 5 | 15.0 (3.9–48.7) | 1.8 (0.8–3.8) |
| Female | 5 | 15.1 (4.6–47.2) | 1.6 (0.7–3.5) |

^a^Some studies reported NCC in study populations consisting exclusively of children or adults.

^b^Values of τ from 0.1 to 0.5 indicate “reasonable” heterogeneity, between 0.5 and 1.0 indicate “fairly high” heterogeneity and above 1.0 indicate “fairly extreme” heterogeneity [118-120].

*BCI* Bayesian credible interval; *n* number of studies; *NCC* neurocysticercosis; *τ* tau-heterogeneity parameter; *%* percentage.

# Table S7. Prevalence of NCC–associated epilepsy among people living in communities

| Country, year(s) of study | Study | Number of people screened for the presence of epilepsy | Prevalence of epilepsy^b^  % (95% *BCI*) | Proportion of NCC among PWE  % (95% *BCI*) | Prevalence of NCC–associated epilepsy in community  %(95% *BCI*) |
| --- | --- | --- | --- | --- | --- |
| Bolivia, 1994 | [3] | 9,955 | 1.3 (1.0–1.5) | 27.9 (20.0–36.9) | 0.3 (0.2–0.5) |
| Burkina Faso, 2007 | [22] | 888 | 7.5 (6.0–9.3) | 21.6 (13.8–30.9) | 2.3 (1.4–3.5) |
| Ecuador, 1992 | [29] | 2,723 | 1.2 (0.8–1.6) | 53.7 (35.3–71.2) | 0.6 (0.4–1.0) |
| Ecuador, 2003 | [34] | 2,415 | 1.0 (0.7–1.5) | 27.9 (11.9–49.3) | 0.3 (0.1–0.6) |
| Honduras, 1997 | [39] | 6,473 | 1.6 (1.3–1.9) | 36.8 (27.4–47.0) | 0.6 (0.4–0.8) |
| India, NA–NA | [47] | 1,028 | 4.9 (3.7–6.3) | 29.4 (19–41.2) | 2.2 (1.4–3.2)^a^ |
| India, NA–NA | [51] | 2,209 | 1.1 (0.7–1.6) | 28.2 (15.1–44.3) | 0.5 (0.2–0.8)^a^ |
| India, NA–NA | [52] | 14,086 | 1.0 (0.8–1.2) | 25.8 (19.8–32.2) | 0.4 (0.3–0.5)^a^ |
| India (urban), 2003–04 | [44] | 38,105 | 0.6 (0.5–0.8) | 18.7 (10.5–29.5) | 0.1 (0.06–0.2) |
| India (rural), 2003–04 | [44] | 12,512 | 0.3 (0.3–0.4) | 34.8 (26.1–44.6) | 0.1 (0.08–0.1) |
| India, 2004–05 | [59] | 1,640 | 5.5 (4.5–6.6) | 31.8 (24.4–40) | 2.7 (2.0–3.6)^a^ |
| India, 2010–11 | [67] | 15,750 | 0.7 (0.6–0.9) | 16.8 (10.7–24.3) | 0.2 (0.1–0.2)^a^ |
| India, 2017–18 | [77] | 59,509 | 0.4 (0.4–0.5) | 12.2 (8.3–16.9) | 0.1 (0.04–0.1) |
| Malawi, 2012–13 | [81] | 69,595 | 0.5 (0.5–0.6) | 3.5 (1.2–7.7) | 0.03 (0.01–0.06) |
| Mozambique, 2007 | [88] | 1,723 | 8.0 (6.9–9.3) | 36.0 (30.1–42.1) | 5.0 (4.0–6.0) |
| Peru, NA–NA | [93] | 576 | 2.6 (1.6–4.1) | 28.5 (10.7–52.5) | 1.2 (0.5–2.4) |
| Peru, 2006–07 | [98] | 1,7450 | 1.7 (1.5–1.9) | 27.8 (23.5–32.4) | 0.7 (0.5–0.8) |
| Uganda, 2009–12 | [106] | 3,8303 | 0.8 (0.7–0.9) | 11.7 (8.5–15.4) | 0.1 (0.08–0.1)^a^ |

^a^Study population included only people with active epilepsy.

^b^Prevalence of epilepsy was not adjusted for sensitivity and specificity of the tool(s) used for epilepsy diagnosis.

*BCI* Bayesian credible interval; *NA* not available; *NCC* neurocysticercosis; *PWE* people with epilepsy; *PWES* people with epileptic seizures; *%* percentage.

# Table S8. Sensitivity analysis results

Posterior median estimates from the Bayesian random effects logistic models (including all 47 studies *with* or *without* formal definition) for uninformative or weakly–informative prior distributions for proportion (*P*) and heterogeneity (*τ*) parameters.

| Prior distribution | | Posterior distribution summary | |
| --- | --- | --- | --- |
| *P* | ***τ*** | **Median *P***  ***%* (95% *BCI*)** | **Median *τ***  **(95% *BCI*)** |
| *Student_t*(3, 0, 2.5)^a^ | *Student_t*(3, 0, 2.5)^a^ | 21.3 (16.3–27.1) | 1.0 (0.8–1.3) |
| *Student_t*(3, 0, 2.5)^a^ | *Half–Cauchy* (0, 0.5) | 21.2 (16.7–26.7) | 1.0 (0.8–1.3) |
| *Logistic*(0, 1) | *Half–Cauchy* (0, 0.5) | 21.5 (16.9–26.8) | 1.0 (0.8–1.3) |
| *Logistic*(0, 1) | *Half–Cauchy* (0, 1) | 21.4 (16.6–26.9) | 1.0 (0.8–1.3) |
| *Logistic*(0, 10) | *Half–Cauchy* (0, 0.5) | 21.1 (16.4–26.6) | 1.0 (0.8–1.3) |
| *Logistic*(0, 10) | *Half–Cauchy* (0, 1) | 21.2 (16.5–26.7) | 1.0 (0.8–1.3) |
| *Normal*(0, 1.81) | *Half–Cauchy* (0, 0.5) | 21.4 (16.7–26.8) | 1.0 (0.8–1.3) |
| *Normal*(0, 1.81) | *Half–Cauchy* (0, 1) | 21.4 (16.8–27.2) | 1.0 (0.8–1.3) |
| *Normal*(0, 1) | *Half–Cauchy* (0, 0.5) | 21.7 (16.9–27.4) | 1.0 (0.8–1.3) |
| *Normal*(0, 1) | *Half–Cauchy* (0, 1) | 21.7 (17.0–27.3) | 1.0 (0.8–1.3) |

^a^The default prior available in *brm()* function. It is an improper flat prior over the reals. *BCI* Bayesian credible interval; *P* proportion; *τ* tau- heterogeneity parameter; *%* percentage.

# Fig S1. Study selection workflow for meta–analysis, subgroup meta–analysis, and meta–regression

*Collectively, these 60 studies were included in the meta–analyses, and the meta–regressions. The orange box shows studies included in the overall meta–analysis for all age groups, combining studies *with* and *without* formal definitions. The green boxes indicate subgroup meta–analyses by study or population characteristics, and the violet box shows the meta–regression. *n* number of studies; *NCC* neurocysticercosis.

# Fig S2. The geographical distribution of the studies on the frequency of NCC among PWES included in the current systematic review

*n* number of studies; *USA* the United States of America

Fig S3. Results of subgroup meta–analyses of the proportion of NCC among people with epileptic seizures (PWES)**.**

***** One study reported the frequency of NCC among people with epilepsy (PWE) and among people with epileptic seizures other than epilepsy (PWES not PWE). *BCI* Bayesian credible interval; *ILAE* International League Against Epilepsy; *n* number of studies; *NCC* neurocysticercosis; *PWE* people with epilepsy; *PWES* people with epileptic seizures; *%* percentage.

# Appendix S1. The Joanna Briggs Institute (JBI) critical appraisal checklist for studies reporting prevalence data

Reviewer_______________________________________Date__________________

Author__________________________Year_________Record Number__________

|  | Yes | No | Unclear | Not applicable |
| --- | --- | --- | --- | --- |
| 1. Was the sample frame appropriate to address the target population? | □ | □ | □ | □ |
| 2. Were study participants sampled in an appropriate way? | □ | □ | □ | □ |
| 3. Was the sample size adequate? | □ | □ | □ | □ |
| 4. Were the study subjects and the setting described in detail? | □ | □ | □ | □ |
| 5. Was the data analysis conducted with sufficient coverage of the identified sample? | □ | □ | □ | □ |
| 6. Were valid methods used for the identification of the condition? | □ | □ | □ | □ |
| 7. Was the condition measured in a standard, reliable way for all participants? | □ | □ | □ | □ |
| 8. Was there appropriate statistical analysis? | □ | □ | □ | □ |
| 9. Was the response rate adequate, and if not, was the low response rate managed appropriately? | □ | □ | □ | □ |

**Overall appraisal:**	Include	□	Exclude	□	Seek further info □

**Comments (Including reason for exclusion)**

____________________________________________________________________________________________________________________________________________________________________________

**Table S9.** The JBI critical appraisal checklist for included studies.

| **Country, year(s) of study** | **Reference** | **Q1** | **Q2** | **Q3** | **Q4** | **Q5** | **Q6** | **Q7** | **Q8** | **Q9** |
| --- | --- | --- | --- | --- | --- | --- | --- | --- | --- | --- |
| Bhutan, 2014–15 | [1] | Yes | Yes | Yes | Yes | Yes | Yes | Yes | N/A | Yes |
| Bolivia, 1994 | [3] | Yes | Yes | Yes | Yes | Yes | Yes | Yes | N/A | Yes |
| Brazil, NA–NA | [6] | Yes | Yes | Yes | Yes | Yes | Yes | Yes | N/A | Yes |
| Brazil, NA–NA | [9] | Yes | Yes | Yes | Yes | Yes | Yes | Yes | N/A | Yes |
| Brazil, 1978–90 | [13] | Yes | Yes | Yes | Yes | Yes | Yes | Yes | N/A | Yes |
| Brazil, 1995–96 | [14] | Yes | No | Yes | Yes | Yes | Yes | Yes | N/A | Yes |
| Brazil, 2005 | [16] | Yes | Yes | Yes | Yes | Yes | Yes | Yes | N/A | Yes |
| Brazil, 2009–11 | [18] | Yes | Yes | Yes | Yes | Yes | Yes | Yes | N/A | Yes |
| Brazil, 2013 | [19] | Yes | Yes | Yes | Yes | Yes | Yes | Yes | N/A | Yes |
| Burkina Faso, 2007 | [22] | Yes | Yes | Yes | Yes | Yes | Yes | Yes | N/A | Yes |
| Cameroon, 1999–05 | [24] | Yes | Yes | Yes | Yes | Yes | Yes | Yes | N/A | Yes |
| Colombia, 1995 | [25] | Yes | Yes | Yes | Yes | Yes | Yes | Yes | N/A | Yes |
| Côte d'Ivoire, 1994–96 | [26] | Yes | Yes | Yes | Yes | Yes | Unclear | Unclear | N/A | Yes |
| Côte d'Ivoire, 2015–17 | [28] | Yes | Yes | Yes | Yes | Yes | Yes | Unclear | N/A | Unclear |
| Ecuador, 1992 | [29] | Yes | Yes | Yes | Yes | Yes | Yes | Yes | N/A | Yes |
| Ecuador, 1986–90 | [33] | Yes | Yes | Yes | Yes | Yes | Unclear | Unclear | N/A | Yes |
| Ecuador, 2003 | [34] | Yes | Yes | Yes | Yes | Yes | Yes | Yes | N/A | Yes |
| Ecuador, 1990–09 | [36] | Yes | Yes | Yes | Yes | Yes | Yes | Yes | N/A | Yes |
| Ecuador, 2012–15 | [37] | Yes | Yes | Yes | Yes | Yes | Yes | Yes | N/A | Yes |
| Honduras, 1997 | [39] | Yes | Yes | Yes | Yes | Yes | Yes | Yes | N/A | Yes |
| Honduras, 1997–05 | [40] | Yes | Yes | Yes | Yes | Yes | Yes | Yes | N/A | Yes |
| India, 1992–93 | [41] | Yes | Yes | Yes | Yes | Yes | Yes | Yes | N/A | Yes |
| India, 1995–99 | [42] | Yes | Yes | Yes | Yes | Yes | Yes | Yes | N/A | Yes |
| India, 1995–01 | [43] | Yes | Yes | Yes | Yes | Yes | Yes | Yes | N/A | Yes |
| India, 2003–04 | [44] | Yes | Yes | Yes | Yes | Yes | Yes | Yes | N/A | Yes |
| India, NA–NA | [46] | Yes | Yes | Yes | Yes | Yes | Yes | Yes | N/A | Yes |
| India, NA–NA | [47] | Yes | Yes | Yes | Yes | Yes | Yes | Yes | N/A | Yes |
| India, NA–NA | [50] | Yes | Yes | Yes | Yes | Yes | Unclear | Unclear | N/A | Yes |
| India, NA–NA | [51] | Yes | Unclear | Yes | Yes | Yes | Yes | Yes | N/A | Yes |
| India, NA–NA | [52] | Yes | Yes | Yes | Yes | Yes | Yes | Yes | N/A | Yes |
| India, NA–NA | [53] | Yes | Yes | Yes | Yes | Yes | Yes | Yes | N/A | Yes |
| India, 1995–01 | [55] | Yes | Yes | Yes | Yes | Yes | Yes | Yes | N/A | Yes |
| India, 2001 | [57] | Yes | Yes | Yes | Yes | Yes | Yes | Yes | N/A | Yes |
| India, 2001–02 | [58] | Yes | Yes | Yes | Yes | Yes | Unclear | Yes | N/A | Yes |
| India, 2004–05 | [59] | Yes | Yes | Yes | Yes | Yes | Yes | Yes | N/A | Yes |
| India, 2006–12 | [61] | Yes | Yes | Yes | Yes | Yes | Yes | Yes | N/A | Yes |
| India, 2006–12 | [63] | Yes | Yes | Yes | Yes | Yes | Yes | Yes | N/A | Yes |
| India, 2009 | [65] | Yes | Yes | Yes | Yes | Yes | Yes | Yes | N/A | Yes |
| India, 2010–11 | [67] | Yes | Yes | Yes | Yes | Yes | Yes | Yes | N/A | Yes |
| India, 2011–12 | [68] | Yes | Unclear | Yes | Yes | Yes | Yes | Yes | N/A | Yes |
| India, 2013–14 | [70] | Yes | Yes | Yes | Yes | Yes | Unclear | Unclear | N/A | Yes |
| India, 2013–16 | [71] | Yes | Yes | Yes | Yes | Yes | Unclear | Unclear | N/A | Yes |
| India, 2015–16 | [73] | Yes | Yes | Yes | Yes | Yes | Unclear | Yes | N/A | Yes |
| India, 2016 | [74] | Yes | Yes | Yes | Yes | Yes | Unclear | Unclear | N/A | Yes |
| India, 2017–18 | [75] | Yes | Yes | Yes | Yes | Yes | Unclear | Unclear | N/A | Yes |
| India, 2017–18 | [77] | Yes | Yes | Yes | Yes | Yes | Yes | Yes | N/A | Yes |
| India, 2017–20 | [78] | Yes | Yes | Yes | Yes | Yes | Unclear | Unclear | N/A | Yes |
| India, 2018–19 | [79] | Yes | Yes | Yes | Yes | Yes | Yes | Yes | N/A | Yes |
| Italy, 2001–16 | [80] | Yes | Yes | Yes | Yes | Yes | Yes | Yes | N/A | Yes |
| Malawi, 2012–13 | [81] | Yes | Yes | Yes | Yes | Yes | Yes | Yes | N/A | Yes |
| Mexico, NA–NA | [82] | Yes | Yes | Yes | Yes | Yes | Unclear | Yes | N/A | Yes |
| Mexico, NA–NA | [83] | Yes | Yes | Yes | Yes | Yes | Yes | Yes | N/A | Yes |
| Mexico, 1989–90 | [85] | Yes | Yes | Yes | Yes | Yes | Unclear | Unclear | N/A | Yes |
| Mexico, 1989–97 | [86] | Yes | Yes | Yes | Yes | Yes | Unclear | Yes | N/A | Yes |
| Mexico, 2000 | [87] | Yes | Yes | Yes | Yes | Yes | Yes | Yes | N/A | Yes |
| Mozambique, 2007 | [88] | Yes | Yes | Yes | Yes | Yes | Yes | Yes | N/A | Yes |
| Namibia, 2012–14 | [90] | Yes | Yes | Yes | Unclear | Yes | Yes | Yes | N/A | Yes |
| Nepal, 2014–16 | [91] | Yes | Yes | Yes | Yes | Yes | Yes | Yes | N/A | Yes |
| Nepal, 2017–18 | [92] | Yes | Yes | Yes | Yes | Yes | Yes | Yes | N/A | Yes |
| Peru, NA–NA | [93] | Yes | Yes | Yes | Yes | Yes | Yes | Yes | N/A | Yes |
| Peru, 1998–99 | [94] | Yes | Yes | Yes | Yes | Yes | Yes | Yes | N/A | Yes |
| Peru, 1999–2000 | [95] | Yes | Yes | Yes | Yes | Yes | Yes | Yes | N/A | Yes |
| Peru, 1999–05 | [97] | Yes | Yes | Yes | Yes | Yes | Yes | Yes | N/A | Yes |
| Peru, 2006–07 | [98] | Yes | Yes | Yes | Yes | Yes | Yes | Yes | N/A | Yes |
| Qatar, 2012–13 | [101] | Yes | Yes | Yes | Yes | Yes | Yes | Yes | N/A | Yes |
| South Africa, 2004–05 | [102] | Yes | Yes | Yes | Yes | Yes | Yes | Yes | N/A | Yes |
| South Africa, 2016–18 | [103] | Yes | Yes | Yes | Yes | Yes | Yes | Yes | N/A | Yes |
| Thailand, 2003–05 | [105] | Yes | Yes | Yes | Yes | Yes | Yes | Yes | N/A | Yes |
| Uganda, 2009–12 | [106] | Yes | Yes | Yes | Yes | Yes | Yes | Yes | N/A | Yes |
| United Republic of Tanzania, 2006 | [108] | Yes | Yes | Yes | Yes | Yes | Yes | Yes | N/A | Yes |
| United Republic of Tanzania, 2009 | [109] | Yes | Yes | Yes | Yes | Yes | Yes | Yes | N/A | Yes |
| United Republic of Tanzania, 2009 | [110] | Yes | Yes | Yes | Yes | Yes | Yes | Yes | N/A | Yes |
| United Republic of Tanzania, 2009 | [111] | Yes | Unclear | Yes | Yes | Yes | Yes | Yes | N/A | Yes |
| United Republic of Tanzania, 2012–13 | [112] | Yes | Yes | Yes | Yes | Yes | Yes | Yes | N/A | Yes |
| United Republic of Tanzania, 2018–20 | [113] | Yes | Yes | Yes | Yes | Yes | Yes | Yes | N/A | Yes |
| USA, 1996–98 | [114] | Yes | Yes | Yes | Yes | Yes | Yes | Yes | N/A | Yes |
| USA, 2013 | [115] | Yes | Yes | Yes | Yes | Yes | Yes | Yes | N/A | Yes |

Q1: Was the sample frame appropriate to address the target population?

Q2: Were study participants sampled in an appropriate way?

Q3: Was the sample size adequate?

Q4: Were the study subjects and the setting described in detail?

Q5: Was the data analysis conducted with sufficient coverage of the identified sample?

Q6: Were valid methods used for the identification of the condition?

Q7: Was the condition measured in a standard, reliable way for all participants?

Q8: Was there appropriate statistical analysis?

Q9: Was the response rate adequate, and if not, was the low response rate managed appropriately?

*NA* not available; *N/A* not applicable; *USA* the United States of America

# Appendix S2. Bayesian meta–regression to estimate the level of reduction of NCC among PWES between the two periods

In subgroup meta–analyses, we observed a reduced proportion of NCC among PWES in period B compared to period A. To assess the magnitude of the reduction between the two periods, we ran a Bayesian mixed–effect logistic regression model. We included other variables such as *Region*, *Imaging–technology* and *Study setting* in the model to assess if these could act as confounder of the effect of the period on the NCC–proportion among PWES. This analysis includes data from only studies *with* formal definitions. Meta–regressions stratified by regions were also run to explore a potential effect modification by region.

**Exposure of interest**

***Period*:** categorical variable with two levels,

- period A (reference category, coded as 0)
- period B (coded as 1)

**Potential confounders**

***Region*:** categorical variable with three levels,

- AFR: African Region (reference category, coded as 0),
- AMR: Region of the Americas (coded as 1)
- SEAR: South–East Asian Region (coded as 2)

***Imaging technology*:** types of brain imaging technology used to diagnose NCC. Categorical variable with three levels,

- CT–scan (reference category, coded as 0),
- MRI (coded as 1)
- Both CT & MRI (coded as 2)

***Study setting*:** categorical variable with two levels,

- Clinic: clinical– or hospital–based studies (reference category, coded as 0)
- Community: community–based studies (coded as 1)

The results of meta-regression models are presented in Table S9 and stratified meta-analysis models in Table S10. For all models, R–hat (Gelman–Rubin statistics) values were below 1.

**Table S10.** All possible meta–regression models with outputs (Prevalence ratio)

| Model | Independent Variables included in the regression model | *PR* (95% *BCI*) | | | | | |
| --- | --- | --- | --- | --- | --- | --- | --- |
|  |  | **Period (ref = A)** | **Region (ref = AFR)** | | **Imaging (ref = CT)** | | **St. setting (ref = Clinic)** |
|  |  | **Period B** | **AMR** | **SEAR** | **MRI** | **CT&MRI** | **Community** |
| 1 | Period | 0.5 (0.3–0.8) |  |  |  |  |  |
| 2 | Period, Region | 0.5 (0.3–0.8) | 1.1 (0.6–1.8) | 1.5 (0.9–2.5) |  |  |  |
| 3 | Period, Imaging–technology | 0.5 (0.3–0.8) |  |  | 1.2 (0.7–1.9) | 1.3 (0.8–2.1) |  |
| 4 | Period, Study–setting | 0.5 (0.3–0.8) |  |  |  |  | 1.4 (1.0–2.0) |

*AFR* African Region; *AMR* Region of the Americas; *BCI* Bayesian credible interval; *CT* computed tomography; *MRI* magnetic resonance imaging; *NCC* neurocysticercosis; *PR* prevalence proportion ratios; *PWES* people with epileptic seizures; *ref* reference category; *SEAR* South–East Asian Region; *%* percentage.

**Table S11.** Proportion of NCC among PWES by period and regions, prevalence proportion ratios between the two periods and % of the prevalence proportion ratios below 1

| Region | Proportion of NCC among PWES | | | A vs B  *PR* (95% *BCI*) | % of *PR* less than 1.0 |
| --- | --- | --- | --- | --- | --- |
|  | **Period A**  **% (95% *BCI*)** | **Period B**  **% (95% *BCI*)** | **Period A & B**  **% (95% *BCI*)** |  |  |
| AFR | 35 (18–57) | 14 (4–44) | 20 (8–40) | 0.4 (0.1–1.5) | 92 |
| AMR | 30 (22–40) | 16 (8–36) | 25 (18–34) | 0.5 (0.3–1.3) | 93 |
| SEAR | 39 (26–54) | 26 (16–41) | 32 (23–43) | 0.7 (0.4–1.2) | 92 |

*AFR* African Region; *AMR* Region of the Americas; *BCI* Bayesian credible interval; *NCC* neurocysticercosis; *PR* prevalence proportion ratios; *PWES* people with epileptic seizures; *SEAR* South–East Asian Region; *%* percentage.

# References

1.	Brizzi K, Pelden S, Tshokey T, Nirola DK, Diamond MB, Klein JP, et al. Neurocysticercosis in Bhutan: A cross-sectional study in people with epilepsy. Trans R Soc Trop Med Hyg. 2016;110:517-26.

2.	Del Brutto OH. Diagnostic criteria for neurocysticercosis, revisited. Pathog Glob Health. 2012;106:299-304.

3.	Nicoletti A, Bartoloni A, Sofia V, Bartalesi F, Chavez JR, Osinaga R, et al. Epilepsy and neurocysticercosis in rural Bolivia: a population‐based survey. Epilepsia. 2005;46:1127-32.

4.	Del Brutto OH, Rajshekhar V, White AC, Jr., Tsang VC, Nash TE, Takayanagui OM, et al. Proposed diagnostic criteria for neurocysticercosis. Neurology. 2001;57:177-83.

5.	the CoCaTo, Epilepsy ILA. Proposal for revised clinical and electroencephalographic classification of epileptic seizures. From the Commission on Classification and Terminology of the International League Against Epilepsy. Epilepsia. 1981;22:489-501.

6.	Arruda WO. Etiology of epilepsy. A prospective study of 210 cases. Arq Neuropsiquiatr. 1991;49:251-4.

7.	Mazer S, Antoniuk A, Ditzel LF, Araujo JC. The computed tomographic spectrum of cerebral cysticercosis. Comput Radiol. 1983;7:373-8.

8.	Minguetti G, Ferreira MV. Computed tomography in neurocysticercosis. J Neurol Neurosurg Psychiatry. 1983;46:936-42.

9.	Secchi TL, Brondani R, Bragatti JA, Bizzi JWJ, Bianchin MM. Evaluating the Association of Calcified Neurocysticercosis and Mesial Temporal Lobe Epilepsy With Hippocampal Sclerosis in a Large Cohort of Patients With Epilepsy. Front Neurol. 2022;12:769356.

10.	Del Brutto OH, Nash TE, White AC, Jr., Rajshekhar V, Wilkins PP, Singh G, et al. Revised diagnostic criteria for neurocysticercosis. J Neurol Sci. 2017;372:202-10.

11.	Scheffer IE, Berkovic S, Capovilla G, Connolly MB, French J, Guilhoto L, et al. ILAE classification of the epilepsies: Position paper of the ILAE Commission for Classification and Terminology. Epilepsia. 2017;58:512-21.

12.	Pack AM. Epilepsy Overview and Revised Classification of Seizures and Epilepsies. Continuum (Minneap Minn). 2019;25:306-21.

13.	Trentin AP, Teive HA, Tsubouchi MH, Paola Ld, Minguetti G. CT findings in 1000 consecutive patients with seizures. Arq Neuropsiquiatr. 2002;60:416-9.

14.	Trevisol-Bittencourt PC, Silva NCd, Figueredo R. Neurocisticercose em pacientes internados por epilepsia no hospital regional de Chapecó regiäo oeste do estado de Santa Catarina. Arq Neuropsiquiatr. 1998;56:53-8.

15.	Sotelo J, Guerrero V, Rubio F. Neurocysticercosis: a new classification based on active and inactive forms: a study of 753 cases. Arch Intern Med. 1985;145:442-5.

16.	Tedrus GMAS, Fonseca LC, Nogueira Junior E. Intracranial calcification suggestive of neurocysticercosis in cranial computed tomography scan: relationship with different clinical conditions. Rev Ciênc Méd. 2007;16:145-50.

17.	Del Brutto OH, Wadia NH, Dumas M, Cruz M, Tsang VC, Schantz PM. Proposal of diagnostic criteria for human cysticercosis and neurocysticercosis. J Neurol Sci. 1996;142:1-6.

18.	Souza-Pereira SRd, Faleiros BE, Coelho BCP, Lara MT, Portela EJ, Teixeira AL. Neurocisticercose em pacientes epilépticos acompanhados em um ambulatório especializado. Rev Bras Neurol. 2011;47:7-10.

19.	de Oliveira Taveira M, Morita ME, Yasuda CL, Coan AC, Secolin R, Luiz Cunha da Costa A, et al. Neurocysticercotic Calcifications and Hippocampal Sclerosis: A Case-Control Study. PLoS One. 2015;10:e0131180.

20.	Fisher RS, Acevedo C, Arzimanoglou A, Bogacz A, Cross JH, Elger CE, et al. ILAE official report: a practical clinical definition of epilepsy. Epilepsia. 2014;55:475-82.

21.	Berg AT, Berkovic SF, Brodie MJ, Buchhalter J, Cross JH, van Emde Boas W, et al. Revised terminology and concepts for organization of seizures and epilepsies: report of the ILAE Commission on Classification and Terminology, 2005-2009. Epilepsia. 2010;51:676-85.

22.	Millogo A, Nitiema P, Carabin H, Boncoeur-Martel MP, Rajshekhar V, Tarnagda Z, et al. Prevalence of neurocysticercosis among people with epilepsy in rural areas of Burkina Faso. Epilepsia. 2012;53:2194-202.

23.	Engel J, Jr. ILAE classification of epilepsy syndromes. Epilepsy Res. 2006;70 Suppl 1:S5-10.

24.	Kuate Tegeu C, Mbahe S, Nguefack S, Bassong PY, Fonsah J, Fogang Y, et al. Aetiologies and risk factors for adult epilepsies: Experience of the Yaoundé central hospital (Cameroon). Epilepsies. 2010;22:74-8.

25.	Palacio LG, Jiménez I, Garcia HH, Jiménez ME, Sánchez JL, Noh J, et al. Neurocysticercosis in persons with epilepsy in Medellin, Colombia. Epilepsia. 1998;39:1334-9.

26.	N'Dri K, Thera M, Bedi MO, Konan A, Ettien F, Zunon-Kipre É, et al. Contribution of computed tomography to the diagnosis of symptomatic epilepsy. Sante. 1999;9:61-4.

27.	Loiseau P, Jallon P. Dictionnaire analytique d'épileptologie clinique: John Libbey Eurotext; 1990. (French)

28.	Soumahoro MK, Melki J, Assi B, Kangah YL, Camara M, Tazemda-Kuitsouc GB, et al. Seroprevalence of cysticercosis among epileptic patients attending neurological units in the urban area of Abidjan. Microorganisms. 2021;9.

29.	Cruz ME, Schantz PM, Cruz I, Espinosa P, Preux P-M, Cruz A, et al. Epilepsy and neurocysticercosis in an Andean community. Int J Epidemiol. 1999;28:799-803.

30.	Kramer LD, Locke GE, Byrd SE, Daryabagi J. Cerebral cysticercosis: documentation of natural history with CT. Radiology. 1989;171:459-62.

31.	Byrd S, Locke G, Biggers S, Percy A. The computed tomographic appearance of cerebral cysticercosis in adults and children. Radiology. 1982;144:819-23.

32.	Commission on Classification and Terminology of the International League Against Epilepsy. Guidelines for epidemiologic studies on epilepsy. Commission on Epidemiology and Prognosis, International League Against Epilepsy. Epilepsia. 1993;34:592-6.

33.	Del Brutto OH, Noboa CA. Late-onset epilepsy in Ecuador: aetiology and clinical features in 225 patients. J Trop Geogr Neurol. 1991;1:31-4.

34.	Del Brutto OH, Santibáñez R, Idrovo L, Rodriguez S, Díaz‐Calderón E, Navas C, et al. Epilepsy and neurocysticercosis in Atahualpa: a door‐to‐door survey in rural coastal Ecuador. Epilepsia. 2005;46:583-7.

35.	Commission on Classification and Terminology of the International League Against Epilepsy. Proposal for revised classification of epilepsies and epileptic syndromes. Commission on Classification and Terminology of the International League Against Epilepsy. Epilepsia. 1989;30:389-99.

36.	Del Brutto OH, Del Brutto VJ. Calcified neurocysticercosis among patients with primary headache. Cephalalgia. 2012;32:250-4.

37.	Del Brutto OH, Arroyo G, Del Brutto VJ, Zambrano M, García HH. On the relationship between calcified neurocysticercosis and epilepsy in an endemic village: A large-scale, computed tomography-based population study in rural Ecuador. Epilepsia. 2017;58:1955-61.

38.	Kimura-Hayama ET, Higuera JA, Corona-Cedillo R, Chavez-Macias L, Perochena A, Quiroz-Rojas LY, et al. Neurocysticercosis: radiologic-pathologic correlation. Radiographics. 2010;30:1705-19.

39.	Medina MT, Durón RM, Martínez L, Osorio JR, Estrada AL, Zúniga C, et al. Prevalence, incidence, and etiology of epilepsies in rural Honduras: the Salama Study. Epilepsia. 2005;46:124-31.

40.	Medina MT, Aguilar-Estrada RL, Alvarez A, Durõn RM, Martínez L, Dubõn S, et al. Reduction in rate of epilepsy from neurocysticercosis by community interventions: The Salamá, Honduras study. Epilepsia. 2011;52:1177-85.

41.	Nair K, Jayakumar P, Taly A, Arunodya G, Swamy H, Shanmugam V. CT in simple partial seizures in children: a clinical and computed tomography study. Acta Neurol Scand. 1997;95:197-200.

42.	Hussain J, Srinivasan S, Serane VT, Mahadevan S, Elangovan S, Bhuvaneswari V. Cranial computed tomography in partial motor seizures. Indian J Pediatr. 2004;71:641-4.

43.	Singh G, Singh P, Singh I, Rani A, Kaushal S, Avasthi G. Epidemiologic classification of seizures associated with neurocysticercosis: observations from a sample of seizure disorders in neurologic care in India. Acta Neurol Scand. 2006;113:233-40.

44.	Rajshekhar V, Raghava MV, Prabhakaran V, Oommen A, Muliyil J. Active epilepsy as an index of burden of neurocysticercosis in Vellore district, India. Neurology. 2006;67:2135-9.

45.	JwAs S. Epidemiology of the epilepsies. J Neurol Neurosurg Psychiatry. 1996;6:433-43.

46.	Singhi S, Singhi P. Clinical profile and etiology of partial seizures in North Indian infants and children. J Epilepsy. 1997;10:32-6.

47.	Devi KR, Borbora D, Upadhyay N, Goswami D, Rajguru SK, Narain K. Neurocysticercosis in patients with active epilepsy in the tea garden community of Assam, Northeast India. Sci Rep. 2021;11:7433.

48.	Garg RK. Diagnostic criteria for neurocysticercosis: some modifications are needed for Indian patients. Neurol India. 2004;52:171-7.

49.	Senanayake N, Roman GC. Epidemiology of epilepsy in developing countries. Bull World Health Organ. 1993;71:247-58.

50.	Ponnatapura J, Vemanna S, Ballal S, Singla A. Utility of Magnetic Resonance Imaging Brain Epilepsy Protocol in New-Onset Seizures: How is it Different in Developing Countries? J Clin Imaging Sci. 2018;8:43.

51.	Raina SK, Razdan S, Pandita KK, Sharma R, Gupta VP, Razdan S. Active epilepsy as indicator of neurocysticercosis in rural northwest India. Epilepsy Res Treat. 2012;2012:802747.

52.	Goel D, Dhanai JS, Agarwal A, Mehlotra V, Saxena V. Neurocysticercosis and its impact on crude prevalence rate of epilepsy in an Indian community. Neurol India. 2011;59:37-40.

53.	Prasad A, Gupta RK, Pradhan S, Tripathi M, Pandey CM, Prasad KN. What triggers seizures in neurocysticercosis? A MRI-based study in pig farming community from a district of North India. Parasitol Int. 2008;57:166-71.

54.	Garcia HH, Del Brutto OH, Nash TE, White AC, Jr., Tsang VC, Gilman RH. New concepts in the diagnosis and management of neurocysticercosis (Taenia solium). Am J Trop Med Hyg. 2005;72:3-9.

55.	Mehta S, Singh G. Electroclinical characteristics of new-onset seizures associated with neurocysticercosis. Neurol India. 2014;62:159-63.

56.	Lüders H, Acharya J, Baumgartner C, Benbadis S, Bleasel A, Burgess R, et al. Semiological seizure classification. Epilepsia. 1998;39:1006-13.

57.	Murthy JMK, Seshadri V. Prevalence, clinical characteristics, and seizure outcomes of epilepsy due to calcific clinical stage of neurocysticercosis: Study in a rural community in south India. Epilepsy Behav. 2019;98:168-72.

58.	Patel NH, Jain AR, Iyer VK, Shah AG, Jain DA, Shah AA. Clinico - diagnostic and therapeutic relevance of computed tomography scan of brain in children with partial seizures. Ann Indian Acad Neurol. 2013;16:352-6.

59.	Prasad KN, Amit P, Gupta RK, Kavindra N, Sunil P, Mukesh T, et al. Neurocysticercosis in patients with active epilepsy from the pig farming community of Lucknow district, north India. Trans R Soc Trop Med Hyg. 2009;103:144-50.

60.	Lwanga SK, Lemeshow S. Sample size determination in health studies: a practical manual: World Health Organization; 1991.

61.	Murthy J, Jaiswal S, Reddy M, Srikrishna S. Incidence Study of Epilepsy using the ILAE 2017 Classification of Epilepsies in a Cohort of School Children Accessing Education in Government Primary Schools in South India. Neurol India. 2020;68:1389-93.

62.	Fisher RS, Cross JH, French JA, Higurashi N, Hirsch E, Jansen FE, et al. Operational classification of seizure types by the International League Against Epilepsy: Position Paper of the ILAE Commission for Classification and Terminology. Epilepsia. 2017;58:522-30.

63.	Jaiswal SK, Murthy JM, Reddy M, Srikrishna S. Incidence of seizures due to degenerative phase of neurocysticercosis: A study in a cohort of primary school children in south india. Neurol Asia. 2019;24:121-5.

64.	Beghi E, Carpio A, Forsgren L, Hesdorffer DC, Malmgren K, Sander JW, et al. Recommendation for a definition of acute symptomatic seizure. Epilepsia. 2010;51:671-5.

65.	Sil A, Das K, Das NK, Chakraborty D, Mazumdar G, Tripathi SK. Use of anti-epileptic drugs in a tertiary care hospital of Eastern India with emphasis on epilepsy due to neurocysticercosis. Indian J Pharmacol. 2012;44:106-10.

66.	Garcı́a HH, Del Brutto OH. Imaging findings in neurocysticercosis. Acta tropica. 2003;87:71-8.

67.	Singh G, Bawa J, Chinna D, Chaudhary A, Saggar K, Modi M, et al. Association between epilepsy and cysticercosis and toxocariasis: A population-based case-control study in a slum in India. Epilepsia. 2012;53:2203-8.

68.	Gowda VKN, Vasanna SH, Pragalath K, Shivananda, Lakskman RR, Premalatha G, et al. Study of etiological profile of infantile and childhood focal seizures at a tertiary care centre in South India. J Pediatr Neurol. 2013;11:235-40.

69.	Rajshekhar V, Haran RP, Prakash GS, Chandy MJ. Differentiating solitary small cysticercus granulomas and tuberculomas in patients with epilepsy. Clinical and computerized tomographic criteria. J Neurosurg. 1993;78:402-7.

70.	Anand NN, Alam KC, Padma V. Clinical and etilogical profile of new onset focal seizures. Int J Pharma Bio Sci. 2015;6.

71.	Samanta M, Mallick AK, Mohanty G, Swain KP. Clinicoradiological evaluation of newly diagnosed epilepsy: A monocentric prospective study from a tertiary care hospital of eastern india. J Clin Diagn Res. 2018;12.

72.	Thurman DJ, Beghi E, Begley CE, Berg AT, Buchhalter JR, Ding D, et al. Standards for epidemiologic studies and surveillance of epilepsy. Epilepsia. 2011;52 Suppl 7:2-26.

73.	Verma M, Kumar C, Alam A, Kumar R, Koonwar S, Gupta S, et al. Role of neuroimaging in children with first unprovoked seizure: A prospective observational study. Epilepsy Behav. 2021;124:108317.

74.	Kankane A, Kankane A. Clinical and demographic profile of person with epilepsy in tertiary care hospital of Bundelkhand region, Central India. J Evol Med Dent Sci. 2018;7:214-7.

75.	Khushbu M, Anshuman S, Utkarsh S, Prasun B. Occurrence of neurocysticercosis in patients presenting with seizure and its radiological evaluation. Int J Med Sci Public Health. 2018;7:605-10.

76.	McAbee GN, Wark JE. A practical approach to uncomplicated seizures in children. Am Fam Physician. 2000;62:1109-16.

77.	Singh G, Singhal S, Paul BS, Bansal N, Chaudhary A, Sharma S, et al. Clinical characteristics of epilepsy in resource-limited communities in Punjab, Northwest India. Epilepsia Open. 2020;5:582-95.

78.	Synmon B, Phukan P, Singh BK, Hussain M, Sharma SR, Hynniewta Y. Structural Brain Lesions in Epilepsy Patients: An Experience from Northeast India. Int J Epilepsy. 2021;7:70-8.

79.	Mital AK, Choudhary P, Jain RB. Prevalence and risk factors for neurocysticercosis in children with a first-onset seizure in rural North India. Paediatr Int Child Health. 2020;40:158-65.

80.	Zammarchi L, Angheben A, Fantoni T, Chiappini E, Mantella A, Galli L, et al. Screening for neurocysticercosis in internationally adopted children: Yield, cost and performance of serological tests, Italy, 2001 to 2016. Euro Surveill. 2018;23.

81.	Keller L, Stelzle D, Schmidt V, Carabin H, Reinhold AK, Keller C, et al. Community-level prevalence of epilepsy and of neurocysticercosis among people with epilepsy in the Balaka district of Malawi: A cross-sectional study. PLoS Negl Trop Dis. 2022;16:e0010675.

82.	Medina MT, Rosas E, Rubio-Donnadieu F, Sotelo J. Neurocysticercosis as the main cause of late-onset epilepsy in Mexico. Arch Intern Med. 1990;150:325-7.

83.	Tapia-Romero R, Romero-Crisóstomo J, García-Rodea R, Meza-Lucas A, Parra-Ortega I, López-Martínez B, et al. Search for antibodies to taenia solium cysticerci in paediatric patients with seizures. Trop Biomed. 2018;35:365-72.

84.	Carpio A, Fleury A, Romo ML, Abraham R, Fandino J, Duran JC, et al. New diagnostic criteria for neurocysticercosis: Reliability and validity. Ann Neurol. 2016;80:434-42.

85.	Aguilar-Rebolledo F, Sosa-Villalobos R, del Castillo-Troncoso C. [Should computed axial tomography of the skull be done in all pediatric patients with epilepsy?]. Bol Med Hosp Infant Mex. 1992;49:845-50.

86.	Rogel-Ortiz FJ. Epilepsy in the adult. Prospective study of 130 cases. Gac Med Mex. 1999;135:363-8.

87.	Suástegui R, Gutierrez J, Ramos R, Bouchan S, Navarrete H, Ruiz J, et al. [Clinical characteristics of the late-onset epilepsy in Mexico to the beginning of the new millennium: 455 cases]. Rev Invest Clin. 2009;61:354-63.

88.	Assane YA, Trevisan C, Schutte CM, Noormahomed EV, Johansen MV, Magnussen P. Neurocysticercosis in a rural population with extensive pig production in Angónia district, Tete Province, Mozambique. Acta Tropica. 2017;165:155-60.

89.	Fisher RS, van Emde Boas W, Blume W, Elger C, Genton P, Lee P, et al. Epileptic seizures and epilepsy: definitions proposed by the International League Against Epilepsy (ILAE) and the International Bureau for Epilepsy (IBE). Epilepsia. 2005;46:470-2.

90.	Segamwenge IL, Kioko NP, Mukulu C, Jacob O, Humphrey W, Augustinus J. Neurocysticercosis among patients with first time seizure in Northern Namibia. Pan Afr Med J. 2016;24:127.

91.	Gupta MM, Chaudhary N, Pathak S, Agrawal N, Yadav J, Shrestha S, et al. Neurocysticercosis in Children with Seizures: A Cross-Sectional Study. Int J Pediatr. 2018;2018:1030878.

92.	Thapa DK, Nepal PR, Karki KT. Prevalence of Neurocysticercosis among Seizure patients:-Single Center Study. East Green Neurosurg. 2020;2:7-12.

93.	Garvey BT, Moyano LM, Ayvar V, Rodriguez S, Gilman RH, Gonzalez AE, et al. Neurocysticercosis among People Living Near Pigs Heavily Infected with Cysticercosis in Rural Endemic Peru. Am J Trop Med Hyg. 2018;98:558-64.

94.	Gaffo A, Guillén-Pinto D, Campos-Olazábal P, Burneo J. Cysticercosis as the main cause of partial seizures in children in Peru. Rev Neurol. 2004;39:924-6.

95.	Montano S, Villaran M, Ylquimiche L, Figueroa J, Rodriguez S, Bautista C, et al. Neurocysticercosis: association between seizures, serology, and brain CT in rural Peru. Neurology. 2005;65:229-33.

96.	Sanders JW, Shorvon SD. The epilepsies. In: Martin CN, Hughes RAC, editors. The epidemiology of neurological disorders London: BMJ Books; 1998.

97.	Villarán MV, Montano SM, Gonzalvez G, Moyano LM, Chero JC, Rodriguez S, et al. Epilepsy and neurocysticercosis: an incidence study in a Peruvian rural population. Neuroepidemiology. 2009;33:25-31.

98.	Moyano LM, Saito M, Montano SM, Gonzalvez G, Olaya S, Ayvar V, et al. Neurocysticercosis as a cause of epilepsy and seizures in two community-based studies in a cysticercosis-endemic region in Peru. PLoS Negl Trop Dis. 2014;8:e2692.

99.	Saavedra H, Gonzales I, Alvarado MA, Porras MA, Vargas V, Cjuno RA, et al. [Neurocysticercosis diagnosis and management in Peru]. Rev Peru Med Exp Salud Publica. 2010;27:586-91.

100.	Garcia HH, Gonzalez AE, Rodriguez S, Tsang VC, Pretell EJ, Gonzales I, et al. Neurocysticercosis: unraveling the nature of the single cysticercal granuloma. Neurology. 2010;75:654-8.

101.	Al-shami R, Khair AM, Elseid M, Ibrahim K, Al-Ahmad A, Elsetouhy A, et al. Neuro-imaging evaluation after the first afebrile seizure in children: A retrospective observational study. Seizure. 2016;43:26-31.

102.	Foyaca-Sibat H, Cowan LD, Carabin H, Targonska I, Anwary MA, Serrano-Ocana G, et al. Accuracy of serological testing for the diagnosis of prevalent neurocysticercosis in outpatients with epilepsy, Eastern Cape Province, South Africa. PLoS Negl Trop Dis. 2009;3:e562.

103.	Mabaso SH, Bhana-Nathoo D, Lucas S. An audit of CT brain findings in adults with new-onset seizures in a resource restricted setting in South Africa. S Afr J Radiol. 2022;26:2294.

104.	Gavvala JR, Schuele SU. New-Onset Seizure in Adults and Adolescents: A Review. JAMA. 2016;316:2657-68.

105.	Phuttharak W, Sawanyawisuth K, Kawiwungsanon A, Tiamkao S. The appropriate neuroimaging study in persons with epilepsy. Neurol Sci. 2011;32:969-71.

106.	Hemmer B, Prazeres da Costa C, Dupont F. Risk factors and socio-economic burden of neurocysticercosis in Uganda: Technical University of Munich; 2018.

107.	Zack MM, Kobau R. National and state estimates of the numbers of adults and children with active epilepsy—United States, 2015. MMWR Morb Mortal Wkly Rep. 2017;66:821-5.

108.	Winkler AS, Blocher J, Auer H, Gotwald T, Matuja W, Schmutzhard E. Epilepsy and neurocysticercosis in rural Tanzania—an imaging study. Epilepsia. 2009;50:987-93.

109.	Hunter E, Burton K, Iqbal A, Birchall D, Jackson M, Rogathe J, et al. Cysticercosis and epilepsy in rural Tanzania: A community-based case-control and imaging study. Trop Med Int Health. 2015;20:1171-9.

110.	Mwanjali G, Kihamia C, Kakoko DV, Lekule F, Ngowi H, Johansen MV, et al. Prevalence and risk factors associated with human Taenia solium infections in Mbozi District, Mbeya Region, Tanzania. PLoS Negl Trop Dis. 2013;7:e2102.

111.	Burton KJ, Rogathe J, Whittaker R, Mankad K, Hunter E, Burton MJ, et al. Epilepsy in Tanzanian children: Association with perinatal events and other risk factors. Epilepsia. 2012;53:752-60.

112.	Schmidt V, O'Hara MC, Ngowi B, Herbinger KH, Noh J, Wilkins PP, et al. Taenia solium cysticercosis and taeniasis in urban settings: Epidemiological evidence from a health-center based study among people with epilepsy in Dar es Salaam, Tanzania. PLoS Negl Trop Dis. 2019;13:e0007751.

113.	Stelzle D, Makasi C, Schmidt V, Trevisan C, Damme Iv, Welte TM, et al. Epidemiological, clinical and radiological characteristics of people with neurocysticercosis in Tanzania- a cross-sectional study. PLoS Negl Trop Dis. 2022;16:e0010911.

114.	Ong S, Talan DA, Moran GJ, Mower W, Newdow M, Tsang VC, et al. Neurocysticercosis in radiographically imaged seizure patients in US emergency departments. Emerg Infect Dis. 2002;8:608.

115.	Saito EK, Mehta B, Wang F, Nakamoto B, McMurtray AM. Headaches More Common among Epilepsy Sufferers with Neurocysticercosis than Other Structural Brain Lesions. Hawaii J Med Public Health. 2017;76:152-5.

116.	Roman G, Sotelo J, Del Brutto O, Flisser A, Dumas M, Wadia N, et al. A proposal to declare neurocysticercosis an international reportable disease. Bull World Health Organ. 2000;78:399-406.

117.	Del Brutto OH, Del Brutto VJ. Reduced percentage of neurocysticercosis cases among patients with late-onset epilepsy in the new millennium. Clin Neurol Neurosurg. 2012;114:1254-6.

118.	Senra H, Gaglianone CG, McPherson S, Unterrainer H. Prevalence of personality disorders in adults with binge eating disorder—A systematic review and Bayesian meta‐analysis. Obes Rev. 2024;25:e13669.

119.	Röver C, Bender R, Dias S, Schmid CH, Schmidli H, Sturtz S, et al. On weakly informative prior distributions for the heterogeneity parameter in Bayesian random‐effects meta‐analysis. Res Synth Methods. 2021;12:448-74.

120.	Reis DJ, Kaizer AM, Kinney AR, Bahraini NH, Holliday R, Forster JE, et al. A practical guide to random-effects Bayesian meta-analyses with application to the psychological trauma and suicide literature. Psychol Trauma. 2023;15:121.
